# Modifiable risk factors in women at high risk of breast cancer: a systematic review

**DOI:** 10.1186/s13058-023-01636-1

**Published:** 2023-04-24

**Authors:** Sarah Y. Cohen, Carolyn R. Stoll, Akila Anandarajah, Michelle Doering, Graham A. Colditz

**Affiliations:** Washington University in St. Louis School of Medicine, St. Louis, MO USA

## Abstract

**Background:**

Modifiable risk factors (alcohol, smoking, obesity, hormone use, and physical activity) affect a woman’s breast cancer (BC) risk. Whether these factors affect BC risk in women with inherited risk (family history, *BRCA1/2* mutations, or familial cancer syndrome) remains unclear.

**Methods:**

This review included studies on modifiable risk factors for BC in women with inherited risk. Pre-determined eligibility criteria were used and relevant data were extracted.

**Results:**

The literature search resulted in 93 eligible studies. For women with family history, most studies indicated that modifiable risk factors had no association with BC and some indicated decreased (physical activity) or increased risk (hormonal contraception (HC)/menopausal hormone therapy (MHT), smoking, alcohol). For women with *BRCA* mutations, most studies reported no association between modifiable risk factors and BC; however, some observed increased (smoking, MHT/HC, body mass index (BMI)/weight) and decreased risk (alcohol, smoking, MHT/HC, BMI/weight, physical activity). However, measurements varied widely among studies, sample sizes were often small, and a limited number of studies existed.

**Conclusions:**

An increasing number of women will recognize their underlying inherited BC risk and seek to modify that risk. Due to heterogeneity and limited power of existing studies, further studies are needed to better understand how modifiable risk factors influence BC risk in women with inherited risk.

**Supplementary Information:**

The online version contains supplementary material available at 10.1186/s13058-023-01636-1.

## Background

A woman’s risk of developing breast cancer is affected significantly by modifiable risk factors, such as alcohol use, smoking, obesity, and physical activity as well as by non-modifiable risk factors, such as *BRCA1* or *BRCA2* mutations, family history of breast cancer, and familial breast cancer syndromes [1, 2]. With initiatives aimed at increasing awareness of family medical history, such as National Family History Day, a growing number of Americans will become aware of a family history of breast cancer [3]. Moreover, as the availability of genetic tests grows and consumers’ views on genetic testing become more favorable, more women will pursue genetic tests to assess cancer risk [4]. Women with a family history of breast cancer, a familial cancer syndrome, or a confirmed breast cancer-linked genetic variant, may seek to modify their risk in non-surgical and non-medical ways.

Many women who discover a family history or obtain genetic testing results will receive very little accompanying guidance on strategies for non-medical risk reduction. Even for those who receive test results from a healthcare professional, such as a geneticist or genetic counselor, the vast majority of these practitioners do not provide advice on modifiable risk factors related to lifestyle [5].

The primary objective of this review was to assess the association of modifiable risk factors and breast cancer in women at high inherited risk for breast cancer and highlight the salient information that should be readily available to the growing numbers of women aware of their breast cancer risk due to genetic testing or elucidation of family history. This information could thus provide guidance to the many women seeking to reduce their elevated inherited risk of breast cancer.

## Methods

This review was performed in accordance with the Preferred Reporting Items for Systematic Reviews and Meta-Analyses (PRISMA) guidelines.

### Search Strategy

For this systematic review, a medical librarian designed and conducted an electronic search using standardized terms and keywords in Ovid Medline, Embase, Scopus, Cochrane Central Register of Controlled Trials, and Clinicaltrials.gov. A full search strategy is included in the additional files (Additional file 6) and all searches took place from June to August 2019. Results were exported to EndNote. The automatic duplicate finder in EndNote was used to eliminate duplicates and the results were also manually searched for duplicates. The identified 7885 articles were screened first at the level of title then abstract, language, and type of work for exclusion. The remaining studies were screened by full text for exclusion. This was performed by a single reviewer (S.C.). Exclusion was determined by the below criteria. The reference lists of included articles were searched by hand for additional citations.

### Eligibility criteria

Eligible studies included published observational studies, including retrospective and prospective case–control and cohort studies evaluating the association of modifiable risk factors on the development of breast cancer in women with a high non-modifiable risk of developing breast cancer. Modifiable risk factors included weight or body mass index (BMI), smoking, alcohol consumption, physical activity, and hormonal contraception (HC) or menopausal hormone therapy (MHT) use. Eligible studies enrolled adults (≥ 18 years old) and identified participants with a non-modifiable risk factor, defined as, first or second-degree family history of breast cancer (FHBC), *BRCA1/2* mutations, or a diagnosed familial cancer syndrome. Only studies published in English were eligible.

Studies were excluded if they did not meet the above eligibility criteria and, additionally, if they did not have a control group that was breast cancer-free and had a positive FHBC, *BRCA1/2* mutation, or a familial cancer syndrome. In addition, studies were excluded if data were not reported in strata defined by family history or genetic marker status. Studies that evaluated other non-modifiable risk factors, including specific non-*BRCA* genetic polymorphisms, or other modifiable risk factors, such as dietary patterns were excluded. In addition, studies were excluded if risk factors were only measured in aggregates, such as the effect of smoking and alcohol but neither individually. Studies involving women that had undergone chemoprevention or prophylactic oophorectomy were excluded. Studies were excluded if they did not use incident breast cancer risk as a primary outcome, including if they measured the outcome of the age of onset of breast cancer. Finally, studies were excluded if only an abstract was available for retrieval.

### Quality

Quality was assessed across the body of evidence based on the aspects of quality used by the Grading of Recommendations, Assessment, Development and Evaluation (GRADE) approach (risk of bias, inconsistency, indirectness, imprecision, and publication bias). One reviewer (S.C.) reviewed all papers for their quality based on the GRADE guidelines.

### Data extraction and analysis

A data extraction form was developed and used for each study to collect any data (confidence intervals and p values) pertaining to the above-specified modifiable risk factors. Study characteristics, sample size, time to follow-up, subjects’ breast cancer type (ductal carcinoma in situ (DCIS) vs. lobular carcinoma in situ (LCIS) vs. ductal carcinoma (DC) vs. lobular carcinoma (LC)) were also extracted from each paper and recorded on the form. Data and study information were extracted by S.C. and all included studies were reviewed at least twice. Each relevant data point was classified based on the type of risk factor exposure (e.g., current, past, adolescent, etc.) as well as based on whether it indicated a significant increase in risk, decrease in risk, or no association with risk of developing breast cancer.

If a study presented both adjusted and unadjusted data, only the most adjusted data were included. For studies that reported data separately on women with 1st- and 2nd-degree FHBC, only data on women with 1st-degree FHBC were included. Finally, if a study separated women with invasive and in situ breast cancer, only data on women with invasive cancer were included.

The significance of the data was determined by the confidence interval (CI) or the p value, if the CI was not given. For data that included multiple levels of a single modifiable risk factor exposure (e.g., increasing glasses of alcohol per day), the p-trend was used to determine significance, if available. Otherwise, the CI of the highest level was included.

After classifying data based on exposure categories and association with breast cancer risk, if an exposure category contained more than one article (before division by subgroup, e.g., *BRCA1* vs. *BRCA2*) it was included in the corresponding bar chart. If two articles used the same study population and reported data on the same exposure, only the article with the larger sample size was included in the chart.

## Results

**Fig. 1:**
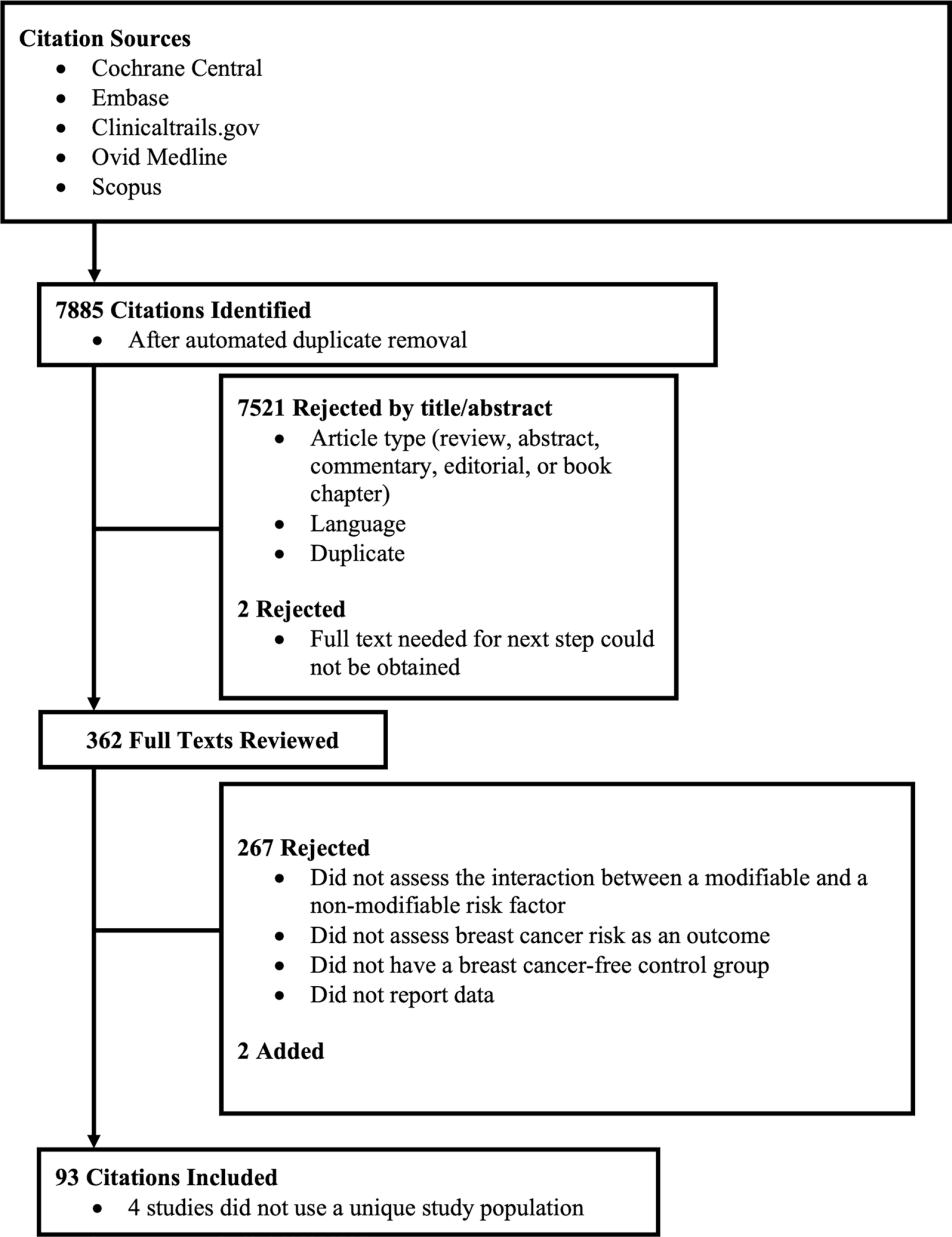
Systematic review chart

The literature search (Fig. 1) identified 7885 citations. In total, 7521 articles were excluded by title or abstract leaving 364 articles for full review. The full text of two of these articles could not be obtained. Of the 362 articles reviewed, 95 studies met the inclusion criteria. Four studies were excluded from the review because they reported on the same exposure in the same study population as other included studies but utilized smaller sample sizes. Two additional studies were added based on screening the citations of relevant articles. Thus, a total of 93 studies were included in the systematic review.

Thirty studies included participants with *BRCA1/2* mutations and 3 additional studies included women with *BRCA1/2* mutations and women with family history. Ten studies were prospective (Table 1) and 23 were retrospective (Table 2). The 33 studies represented 27 distinct study populations and none of the included studies using the same study population reported on the same risk factor.

**Table 1:** Characteristics of included studies on BRCA in prospective studies

Author	Sample size	Alcohol	Smoking	MHT/HC	BMI/Weight	Physical activity	Notes
Cybulski et al. [8]	2498 (213 cases) BRCA1 569 (46 cases) BRCA2	✓					All cases prospectively ascertained
Kim et al. [37]	2737 (242 cases) BRCA1 756 (61 cases) BRCA2				✓- 0.49 (0.30–0.82) (adolescent, BRCA1/2)		All cases prospectively ascertained
Ko et al. [22]	3920 BRCA1 or 2 (544 cases with BRCA1, 153 cases with BRCA 2)		✓ + 1.28 (1.00–1.64) (current, BRCA1/2) 1.34 (1.04–1.73) (duration, BRCA1/2)				All cases prospectively ascertained
Schrijver et al. [38]	2276 (269 cases) BRCA1 1610 (157 cases) BRCA2			✓ + 1.75 (1.03–2.97) (HC, ever, BRCA2)			All cases prospectively ascertained
Kehm et al. [39]	659 (110 cases) BRCA1 526 (69 cases) BRCA2					✓- 0.41 (0.20–0.83) (current, BRCA2)	All cases prospectively ascertained
Lecarpentier et al. [40] *	863 (332 cases) BRCA1 474 (167 cases) BRCA2			✓ + 2.29 (1.07–4.89) (MHT, duration, BRCA1/2)	✓ + − 5.46 (2.49–12.0) (low, BRCA2) 0.56 (0.37–0.85) (high, BRCA1) 0.48 (0.24–0.97) (high, BRCA2)		
Pijpe et al. [41]*	1026 (468 cases) BRCA1/2					✓− 0.63 (0.44–0.91) (adult, BRCA1/2)	
Qian et al. [42]*	14,676 (7360 cases) BRCA1 7912 (4091 cases) BRCA2				✓− 0.83 (0.76–0.90) (adolescent, BRCA1/2) 0.94 (0.90–0.98) (current, BRCA1/2)		
Brohet et al. [43]*	1181 (597 cases) BRCA1 412 (249 cases) BRCA2			✓ + 1.47 (1.16–1.87) (HC, ever, BRCA1/2)			
Lecarpentier et al. [11]*	863 (332 cases) BRCA1 474 (163 cases) BRCA2	✓	✓				

*Secondary analysis of prospectively collected data

✓Study presented data on the association of the modifiable risk factor with breast cancer. Association presented was positive (+), negative (−), or not significant (no ± listed). Associations could be both (+) and (−) if multiple associations were presented

Significant risk estimates (RR/OR/HR (95% CI)) from studies are listed. Results from studies reporting only p values or other measures that did not indicate magnitude of effect are not included in this table

**Table 2:** Characteristics of included studies on BRCA in retrospective studies

Author	Sample size	Alcohol	Smoking	MHT/HC	BMI/Weight	Physical activity
Dennis et al. [6]	2960 (1480 cases) BRCA1 890 (445 cases) BRCA2	✓− 0.82 (0.70–0.96) (current, BRCA1) 0.64 (0.47–0.87) (wine, BRCA1)				
Ghadiriani et al. [25]	1612 (806 cases) BRCA1 582,291 (291,582 cases) BRCA2		✓			
McGuire et al. [7]	497 (195 cases) BRCA1 307 (128 cases) BRCA2	✓− 0.66 (0.45–0.97) (ever, BRCA2)				
Kotsopoulos et al. [44]	864 (432 cases) BRCA1			✓ + − 1.18 (1.03–1.36) (HC, ever, BRCA1) 1.22 (1.04–1.49) (HC, > 5y, BRCA1) 1.45 (1.20–1.75) (HC, early use, BRCA1) 0.80 (0.66–0.97) (HC, current, BRCA1)		
Lee et al. [45]	538 (94 cases) BRCA1/2			✓		
Park et al. [46]	222 (168 cases) BRCA1 359 (250 cases) BRCA2			✓		
Toss et al. [47]	113 (45 cases) BRCA1/2			✓		
Eisen et al. [48]	472 (236 cases) BRCA1			✓ + − 0.58, (0.35–0.96) (MHT, ever, BRCA1) 0.51 (0.27–0.98) (MHT, type, BRCA1)		
Gronwald et al. [27]	696 (348 cases) BRCA1		✓	✓		
Haile et al. [49]	497 (195 cases) BRCA1 307 (128 cases) BRCA2			✓ + − 2.20 (1.26–3.85) (HC, age at first use, BRCA2) 3.46 (2.10–5.70) (HC, before pregnancy, BRCA2) 0.63 (0.42–0.95) (HC, past use, BRCA1) 0.42 (0.20–0.85) (HC, age at first use, BRCA1)		
Heimdal et al. [50]	98 (27 cases) BRCA1			✓		
Brunet et al. [26]	286 (143 cases) BRCA1 86 (43 cases) BRCA2		✓− 0.49 (0.28–0.85) (pack years, BRCA1/2)		✓	
Ginsburg et al. [23]	3840 (1920 cases) BRCA1 1236 (618 cases) BRCA2		✓ + − 1.27 (1.06–1.50) (ever, BRCA1) 0.71 (0.5–1.0) (current, BRCA2)			
Grill et al. [9]	68 (46 cases) BRCA1/2	✓	✓−	✓	✓	✓ +
Kotsopoulos et al. [51]	1594 (797 cases) BRCA1 556 (278 cases) BRCA2				✓− 0.66 (0.46–0.93) (loss, BRCA1/2)	
Lammert et al. [52]	686 (343 cases) BRCA1 200 (100) BRCA2				✓ + 1.72 (1, 08, 2.70) (current, PRM)	✓
Manders et al. [53]	861 (170 cases) BRCA1 265 (48 cases) BRCA2				✓ + 2.10 (1.23–3.59) (weight, POM)	
Nkondjock et al. [10]	137 (89 cases) BRCA1/2	✓	✓		✓ + 4.64 (1.52–14.12) (gain)	✓
Whittemore et al. [24]	497 (195 cases) BRCA1 307 (128 cases) BRCA2		✓ + 2.08 (1.41–3.06) (current, BRCA1/2)			
Narod et al. [54]	1962 (981 cases) BRCA1 660 (330 cases) BRCA2			✓ + 1.18 (1.01–1.38) (HC, ever BRCA1) 1.59 (1.30–1.94) (HC, time since use, BRCA1) 1.02 (1.00–1.03) (HC, duration, BRCA1) 1.42, (1.17–1.75) (HC, use before 1975, BRCA1)		
Bernholtz et al. [55]	776 (403 cases) BRCA1/2			✓ + 1.84 (1.47–2.31) (HC, ever, BRCA1/2)		
Kotsopoulos et al. [56]	4984 (2492 cases) BRCA1			✓ + − 1.18 (1.03–1.36) (HC, ever, BRCA1) 1.22 (1.04–1.49) (HC, duration, BRCA1) 0.80, 0.66–0.97) (HC, current BRCA1)		
Grandi et al [57]	113 BRCA1 or 2 36.9% of BRCA1 and 42.0% of BRCA2 carriers were cases			✓		

✓Study presented data on the association of the modifiable risk factor with breast cancer. Association presented was positive (+), negative (−), or not significant (no ± listed). Associations could be both (+) and (−) if multiple associations were presented

Significant risk estimates (RR/OR/HR (95% CI)) from studies are listed. Results from studies reporting only *p* values or other measures that did not indicate magnitude of effect are not included in this table

Sixty studies included women with a positive family history of breast cancer (FHBC) and 3 additional studies included women with *BRCA1/2* mutations and women with FHBC for a total of 63 studies. Twenty-five studies were prospective (Additional file 4: Table S1), and 38 studies were retrospective (Additional file 5: Table S2). The sixty-three studies represented fifty-two study populations. Included studies that used the same study populations investigated different modifiable risk factors or different categories of exposure for the same modifiable risk factor (e.g., alcohol/day vs. lifetime). Of the 12 studies looking at BMI/weight in women with FHBC, 3 studies used the same cohort; of the 32 studies looking at MHT/HC in women with FHBC, 2 pairs and 1 triplet of studies used the same cohorts; and of the 10 studies looking at alcohol in women with FHBC, 2 studies used the same study population.

**Fig. 2:**
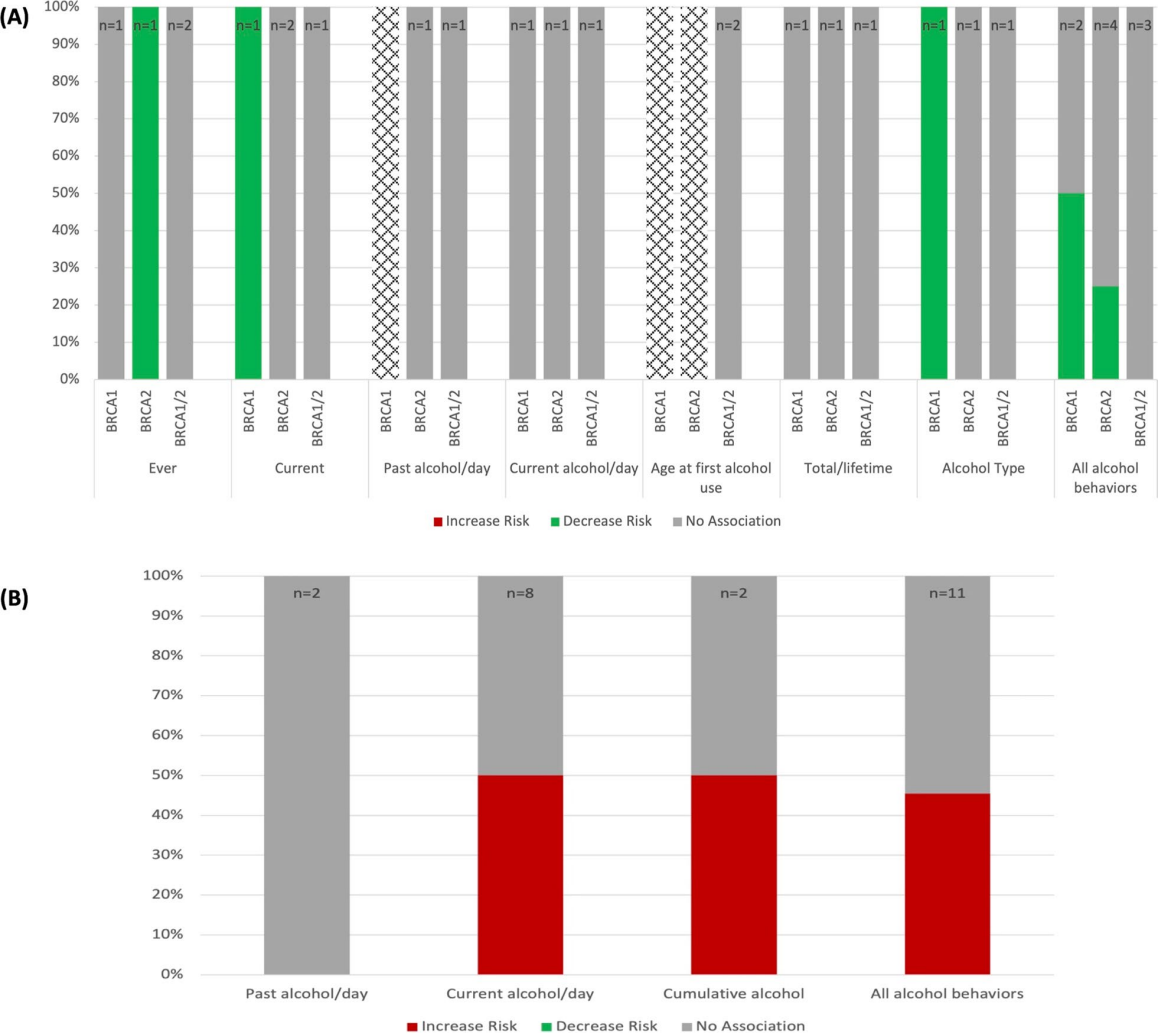
Alcohol and breast cancer risk in women with *BRCA* mutations (*n* = 6) and family history (*n* = 9). Each bar is divided based on the proportion of included studies that demonstrated an increased risk, decreased risk, or no association with risk of breast cancer due to the specified alcohol exposure. Within each alcohol exposure, each study is represented only once. However, because the category “all alcohol behaviors” combines the results of all other exposure categories, studies may be represented more than once, if the results differ by exposure (e.g., decreased risk with current and no association with total/lifetime alcohol consumption). Numbers on the “all alcohol behaviors” bars indicate the range of risk estimates from studies when reported as a ratio measure (OR/RR/HR). Results from studies reporting only p values or other measures that did not indicate magnitude of effect are not included in these ranges. **A** Each bar in the figure represents all of the included studies (*n* = total number of studies) that reported results on the specified alcohol exposure in women with *BRCA* mutations, separated by *BRCA* mutation, if provided. Of the studies assessing alcohol type, one looked at wine drinking and the other did not specify the type of alcohol assessed. Please see Tables 1 and 2 for all studies cited. **B** Each bar in the figure represents all of the included studies (*n* = total number of studies) that reported results on the specified alcohol exposure in women with family history. Please see Additional file 4: Tables S1 and Additional file 5: Table S2 for all studies cited

**Fig. 3:**
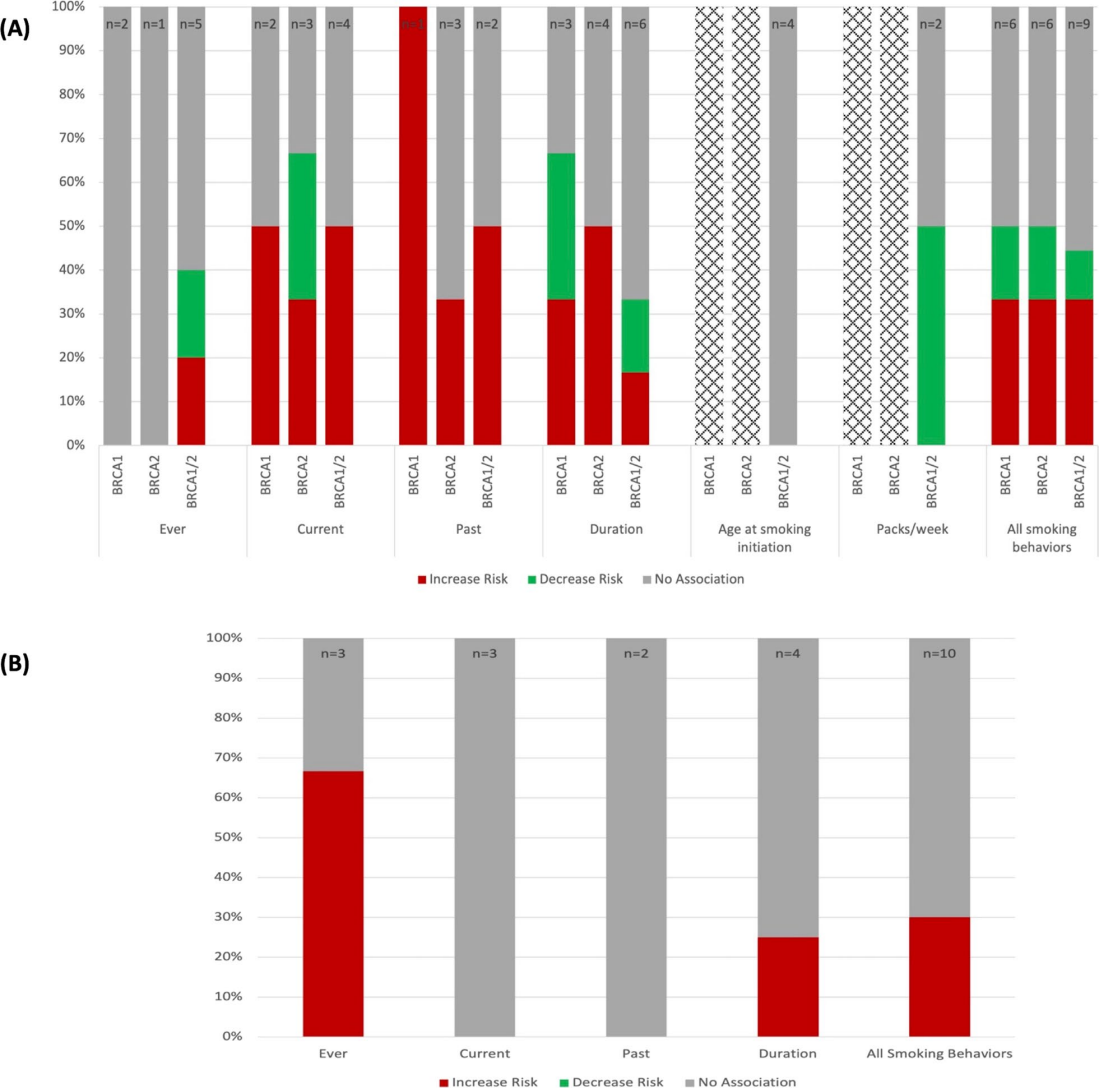
Smoking and breast cancer risk in women with *BRCA* mutations (*n* = 9) and family history (*n* = 9). Each bar is divided based on the proportion of included studies that demonstrated an increased risk, decreased risk, or no association with risk of breast cancer due to the specified smoking exposure. Within each smoking exposure, each study is represented only once. However, because the category “all smoking behaviors” combines the results of all other exposure categories, studies may be represented more than once, if the results differ by exposure (e.g., increased risk with current smoking and no association with past smoking). Numbers on the “all smoking behaviors” bars indicate the range of risk estimates from studies when reported as a ratio measure (OR/RR/HR). Results from studies reporting only p values or other measures that did not indicate magnitude of effect are not included in these ranges. **A** Each bar in the figure represents all of the included studies (*n* = total number of studies) that reported results on the specified smoking exposure in women with BRCA mutations, separated by *BRCA* mutation, if provided. Please see Tables 1 and 2 for all studies cited. **B** Each bar in the figure represents all of the included studies (*n* = total number of studies) that reported results on the specified smoking exposure in women with family history. Please see Additional file 4: Table S1 and Additional file 5: Table S2 for all studies cited

Six studies investigated the effect of alcohol consumption on breast cancer risk in *BRCA 1/2* mutation carriers (Fig. 2A), 10 alcohol consumption in women with FHBC (Fig. 2B), 9 smoking in *BRCA 1/2* mutation carriers (Fig. 3A), 9 smoking in women with FHBC (Fig. 3B), 16 hormonal contraception or menopausal hormone therapy in *BRCA 1/2* mutation carriers (Additional file 1: Figure S1A and B), 32 hormonal contraception or menopausal hormone therapy in women with FHBC (Additional file 1: Figure S1C), 9 BMI or weight in *BRCA 1/2* mutation carriers (Additional file 2: Figure S2A), 12 BMI or weight in women with FHBC (Additional file 2: Figure S2B), 5 physical activity in *BRCA 1/2* mutation carriers (Additional file 3: Figure S3A), and 16 physical activity in women with FHBC. Several of these studies included more than one modifiable risk factor.

### Alcohol consumption in women with BRCA 1/2 mutations

Figure 2A includes data on the association between alcohol consumption and breast cancer (BC) in women with *BRCA* mutations. The data are divided into categories for ever, current, lifetime, age at first alcohol use, current alcohol/day, past alcohol/day, alcohol type, and a combined measure based on these seven exposure categories. Drinks/week was investigated in one study and found to be associated with a decreased risk of BC in women with *BRCA1* mutations and no association with BC risk in women with *BRCA2* mutations [6]. Of the six studies that looked at alcohol and BC risk, two found that a few of the alcohol exposures investigated decreased risk and the remaining studies found no association with BC risk [6–11].

Although the alcohol exposures investigated by each study were organized into a few categories, within those categories there were still multiple definitions of these exposures, as can be seen in Table 3. Ever and current alcohol consumption was defined consistently across studies; however, the other exposures varied.

**Table 3:** Variations in the definitions of alcohol exposures

Total	Nonuser vs. 1-29y vs. > 29y	Never vs. ≤ 780 vs. 781–1404 vs. 1405–2366 vs. > 2366 cups		
Age at first use	< 17yo	> 20yo	Prior to vs. after first full-term birth	Mean age
Current alcohol/day	Nonusers vs. 1–4 vs. > 4 g/day	< 1.7 vs. ≤ 7.9 and > 1.7 vs. > 7.9 g/day		
Past alcohol/day	More than 1 cl glass/day	1–5 vs. 6–10 vs. > 10 glasses/week		
Alcohol type	Wine-exclusive	Wine, spirits, beer		

### Alcohol consumption in women with family history of breast cancer

Figure 2B represents data on the impact of alcohol consumption on the development of BC in women with FHBC, divided into categories for past alcohol/day, current alcohol/day, cumulative alcohol, and all alcohol behaviors, a combined measure based on the other three categories. Additional alcohol exposures that appeared in only one article and thus are not presented in Fig. 2B include duration of alcohol use, age at first alcohol consumption, and current alcohol use (with no indication of quantity per day), which all had no association with BC risk as well as current or ever binging, blacking out, or drinking to the point of hurting one’s health, which all increased risk of BC [12, 13]. Overall, all studies demonstrated that alcohol consumption increased or had no association with BC risk (see Additional file 4: Table S1 and Additional file 5: Table S2 and Fig. 2B) [14–21].

Although the alcohol exposures investigated by each study were organized into a few categories, within those categories the definitions of these exposures still vary significantly as illustrated in Table 4. Table 4 demonstrates the various ways the particular alcohol exposure was defined by the studies included.

**Table 4:** Variations in the definitions of alcohol exposures

Past alcohol/day	During 20’s: 0 vs. < 6 vs. 6–18 vs. 19–32 vs. > 32 g/day	Formerly < 1 vs. 1–1.9 vs. > 2 drinks/day			
Total/Lifetime	< 60 vs. 60–229 vs. > 230 drinks/year	Cumulative grams of alcohol pre-menopause			
Current alcohol/day	Recent 0 vs. < 6 vs. 6–18 vs. 19–32 vs. > 32 g/day	Current < 1 vs. 1–1.9 vs. > 2 drinks/day	0 vs. 1 vs. 1 < 2 vs. 2–3 vs. > 3 drinks/day	Less than weekly vs. weekly vs. daily	< 3 vs. ≥ 3 glasses/day

### Cigarette smoking in women with BRCA 1/2 mutations

Figure 3A depicts data on the association of cigarette smoking with BC risk in women with *BRCA* mutations. Data are separated into exposure categories: ever, current, past, age at smoking initiation, pack-years, and packs/week. Additional exposures that appeared in single studies included total years of smoking, which was associated with increased risk of BC and time since last cigarette use, which found no association with BC [11, 22]. Overall, two studies reported that a few of the smoking exposures were associated with decreased risk of BC and the remaining studies had no association with BC risk [9–12, 23–27].

Analogous to alcohol exposures, smoking exposure definitions also varied across studies as demonstrated in Table 5. The definitions of ever, current, and past were largely consistent across studies, and the remaining exposure categories varied widely.

**Table 5:** Variations in the definitions of smoking exposures

Total/Lifetime	≤ 8 vs. 8 < y ≤ 18 vs. > 18y						
Age at first use	Mean age	Prior to first full-term pregnancy	Within 5 years of menarche	Never vs. ≤ 18 vs. > 18yo	≥ 20 vs. 18–19 vs. ≤ 17yo		
Pack-years	0 vs. ≤ 14 and > 0 vs. > 14py	0–5 vs. 6–20 vs. ≥ 21py	Nonsmoker vs. < 20 vs. ≥ 20py	≤ 2.3 vs. 2.3 < py ≤ 9.8 vs. > 9.8py	0 < py ≤ 5 vs. 5 > py ≤ 10 vs. 10py	0 vs. > 0 ≤ 4 vs. > 4py	0 vs. 1–4 vs. ≥ 5py
Packs/week	< 2 vs. ≤ 2 packs < 5 vs. 5 ≤ packs	0 vs. > 0 < 5 vs. ≥ 5 packs/week					

### Cigarette smoking in women with family history of breast cancer

Figure 3B represents data on the association between cigarette smoking and the development of BC in women with FHBC, divided into ever, current, past, age at initiation of, and duration of smoking as well as a combined measure based on the other four categories. One study additionally looked at cigarettes/day and age at first cigarette use and found no association for either with BC risk [28]. All studies reported that smoking increased or had no association with BC risk (see Additional file 4: Tables S1 and Additional file 5: Table S2 and Fig. 3B) [19, 20, 28–34].

Similar to alcohol exposures, although study findings were organized into categories of smoking exposure, the definitions of some of these exposures remain varied, as shown in Table 6. Ever, current, and past smoking were defined similarly across studies with no specification beyond those labels, whereas duration and age at smoking initiation varied.

**Table 6:** Variations in the definitions of smoking exposures

Age at smoking initiation	Never vs. < 20yo vs. 20 + yo	Never vs. before or < 5 years after menarche vs. 5 + years after menarche	Unspecified		
Cigarettes/day	Never vs. 1–4 vs. 5 +	Unspecified			
Duration (years or pack-years)	Never vs. 1-9y vs. 10 + y	Never vs. 20 + y	0y vs. < 11y vs. 11-20y vs. 21-30y vs. > 30y	Never vs. ≤ 30py vs. > 30py	Unspecified

#### Menopausal hormone therapy, hormonal contraception, weight, and physical activity

Tables 3, 4, 5, and 6 illustrate the myriad ways that exposure to a modifiable factor was defined in the included studies. Although not featured in a table format, this variability was only greater in the remaining modifiable risk factors (MHT/HC, BMI/weight, and physical activity).

### Menopausal hormone therapy and hormonal contraception in women with BRCA 1/2 mutations

Data on the association of MHT/HC with BC risk in women with *BRCA* mutations are included in Additional file 1: Figures S1A, B. Most studies indicated no association with BC, a few indicated an increased risk for MHT/HC use in women with *BRCA1*, and a little less than one-third indicated a decreased risk of BC.

### Menopausal hormone therapy and hormonal contraception in women with family history of breast cancer

Data on the association of MHT/HC with BC risk in women with FHBC are included in Additional file 1: Figure S1C. Most studies indicated no association with BC, a few indicated an increased risk, and one indicated a decreased risk of BC.

### BMI/weight in women with BRCA 1/2 mutations

Data on the association of BMI/weight with BC risk in women with *BRCA* mutations are included in Additional file 2: Figure S2A. Most studies that looked at the relationship between elevated BMI/weight and pre- or post- menopausal BC showed no association, a few studies reported increased risk, and a few decreased risk.

### BMI/weight in women with family history of breast cancer

Data on the association of BMI/weight with BC risk in women with FHBC are included in Additional file 2: Figure S2B. Most studies that looked at the relationship between elevated BMI/weight and pre-menopausal BC showed no association, one reported increased risk and one decreased risk. A little over half of the studies that looked at postmenopausal BC showed an increased risk, two studies showed decreased risk, and the remaining studies showed no association.

### Physical activity in women with BRCA 1/2 mutations

Data on the association of physical activity with BC risk in women with *BRCA* mutations are included in Additional file 3: Figure S3A. All studies that investigated the association between physical activity and BC showed that increased physical activity decreased or had no association with the risk of BC.

### Physical Activity in women with family history of breast cancer

Data on the association of physical activity with BC risk in women with FHBC are included in Additional file 3: Figure S3B. All studies that investigated the association between physical activity and BC showed that increased physical activity decreased or had no association with the risk of BC.

#### Other non-modifiable risk factors

Only one eligible study included participants with a familial cancer syndrome (Li Fraumeni). The study explored the effect of hormonal contraception on breast cancer risk and found that the use of oral contraception had no impact on risk, however, increasing the duration of use increased risk.

#### Quality

The quality of the studies included in this review was analyzed using the GRADE approach, which includes an assessment of risk of bias, inconsistency, indirectness, imprecision, and publication bias. We also assessed the racial/ethnic distribution of the study populations used in included studies to assess the generalizability of findings (Table 7).

**Table 7:** Racial/ethnic distribution of study populations used in included studies

Author	Race/ethnicity data
*BRCA studies*	
Cybulski et al. [8]	NR. Study subjects from 12 countries
Dennis et al. [6]	Ethnicity. Controls: French-Canadian 133 (6.9%), Jewish 359 (18.7%), Other 31 (1.6%), Other White 1402 (72.8%). Cases: French-Canadian 150 (7.8%), Jewish 284 (14.8%), Other 55 (2.9%), Other White 1436 (74.6%). Study subjects from 8 countries
Ghadiriani et al. [25]	NR. Study subjects from 11 countries
McGuire et al. [7]	Study subjects were non-Hispanic white women. Involved institutions from US, Canada, and Australia
Kotsopoulos et al. [44]	Ethnicity. Controls: Other white 367 (85.0%), Jewish 51 (11.8%), French-Canadian 9 (2.1%), Other 5 (1.2%). Cases: Other white 350 (81.0%), Jewish 53 (12.3%), French-Canadian 13 (3.0%), Other 16 (3.7%). Study subjects from 13 countries
Lee et al. [45]	Race and ethnic origin. Controls: White 409 (92%), African-American 35 (8%), not Ashkenazi Jewish 398 (90%), are Ashkenazi Jewish 46 (10%). Cases who are BRCA1/2 mutation carriers: White 88 (94%), African-American 6 (6%), not Ashkenazi Jewish 69 (73%), are Ashkenazi Jewish 25 (27%). Cases who are BRCA1/2 mutation noncarriers: White 1239 (90%), African-American 136 (10%), not Ashkenazi Jewish 1222 (89%), are Ashkenazi Jewish 153 (11%). Study conducted in US
Park et al. [46]	NR, but study conducted in Korea
Toss et al. [47]	NR, but study conducted in Italy
Eisen et al. [48]	Ethnicity. Controls: Other white 233 (82%), Jewish 53 (14%), French-Canadian 15 (3%), Other 2 (1%). Cases: Other white 183 (73%), Jewish 40 (17%), French-Canadian 10 (4%), Other 3 (1%). Subjects from 9 countries
Gronwald et al. [27]	NR, but study conducted in Poland
Haile et al. [49]	Eligible subjects were White non-Hispanic women. Sources from US, Canada and Australia
Heimdal et al. [50]	NR, but study conducted in Norway
Brunet et al. [26]	NR. Study conducted in North America (women resided in either Canada or US)
Ginsburg et al. [23]	NR. Study subjects from 11 countries
Grill et al. [9]	NR but study conducted in Germany
Kim et al. [37]	NR. Study subjects from 17 countries
Ko et al. [22]	NR. The study population was selected from a multicenter longitudinal cohort of BRCA1 and BRCA2 mutation carriers from 80 participating centers in 17 countries including North America, Europe, Asia, the Caribbean, and Latin America
Kotsopoulos et al. [51]	NR. Study subjects from 5 countries
Lammert et al. [52]	NR. Study subjects from 17 countries
Lecarpentier et al. [40]	NR but study conducted in France
Manders et al. [53]	NR but study conducted in the Netherlands
Nkondjock et al. [10]	Participants were French-Canadian. Study conducted in Canada
Pijpe et al. [41]	NR but study conducted in the Netherlands
Qian et al. [42]	Ethnicity. BRCA1 carriers: Caucasian not otherwise specified 13,435 (91.5%). Ashkenazi Jewish 1241 (8.5%). BRCA2 carriers: Caucasian not otherwise specified 7126 (90.1%). Ashkenazi Jewish 786 (9.9%). International study with multiple countries of enrollment
Whittemore et al. [24]	Eligible subjects were non-Hispanic white women. Study subjects from US, Canada, Australia, and New Zealand
Narod et al. [54]	Ethnicity. Controls: Black 14 (1.1%), French-Canadian 97 (7.4%), Jewish 391 (29.8%), Other non-whites 6 (0.5%), Other whites 801 (61.1%), Missing 2 (0.1%). Cases: Black 28 (2.1%), French-Canadian 99 (7.6%), Jewish 414 (31.6%), Other non-whites 11 (0.8%), Other whites 754 (57.4%), Missing 5 (0.4%). Study subjects from 11 countries
Bernholtz et al. [55]	All participants were Jewish. Study conducted in Israel
Brohet et al. [43]	NR. International cohort representing many European countries and Canada
Kotsopoulos et al. [56]	Ethnicity. Controls: French-Canadian 86 (3.5%), Jewish 419 (16.8%), Other white 44 (1.8%). Cases: French-Canadian 97 (3.9%), Jewish 372 (14.9%), Other white 87 (3.5%). Study subjects from 13 countries
Schrijver et al. [38]	NR. Combined multiple international cohort studies conducted in Western countries
Lecarpentier et al. [11]	NR but study conducted in France
Kehm et al. [39]	Race and ethnicity reported by quintiles of age-adjusted baseline recreational physical activity. Q1: non-Hispanic White 2350 (75.4%), other 745 (23.9%), missing 23 (0.7%). Q2: non-Hispanic white 2451 (78.9%), other 626 (20.2%), missing 30 (1.0%). Q3: non-Hispanic white 2506 (80.5%), other 582 (18.7%), missing 27 (0.9%). Q4: non-Hispanic white 2426 (78.1%), other 652 (21.0%), missing 29 (0.9%). Q5: non-Hispanic white 2418 (77.9%), other 662 (21.3%), missing 23 (0.7%). Used data from studies from US, Canada, Australia, and New Zealand
Grandi et al. [57]	NR but study conducted in Italy
*Family history of breast cancer studies*	
Bernstein et al. [58]	Race. Cases: White 2933 (64.6%), Black 1605 (35.4%). Controls: White 3003 (64.6%), Black 1656 (35.4%). Study conducted in US
Gong et al. [59]	All women were African-American. Study conducted in US
Hirose et al. [60]	NR, but study was conducted in Japan
Marchbanks et al. [61]	Race. Cases: white race 2953 (64.5%), black race 1622 (35.5%). Controls: white race 3021 (64.5%), black race 1661 (35.5%). Study conducted in US
Nichols et al. [62]	94.8% white (1799 cases, 7605 controls). Study conducted in US
Nyante et al. [31]	NR. Study conducted in US
Patel et al. [63]	Race. Cases: White 475 (83.4%), Black 92 (16.2%). Controls: White 364 (59.1%), Black 252 (40.9%). Study conducted in US
Reynolds et al. [32]	The cohort is predominantly non-Hispanic white (87%). Study conducted in US
Sprague et al. [64]	NR. Study conducted in US
Murray et al. [65]	Race. First-degree family history cases: White 88.1%, Hispanic 2.5%, Black 6.5%, Other 2.9%. First-degree family history controls: White 87.8%, Hispanic 1.5%, Black 8.7%, Other 2.0%. Second-degree family history cases: White: 90.0%, Hispanic: 1.3%, Black 7.5%, Other 1.3%. Second-degree family history controls: White 90.9%, Hispanic 1.9%, Black 6.4%, Other 0.9%. Study conducted in US
Newcomb et al. [66]	NR. Study conducted in US
Silvera et al. [67]	NR. Study conducted in Canada
Bardia et al. [68]	NR. Study conducted in US
Brinton et al. [30]	NR. Study conducted in US
Colditz et al. [20]	NR. Study conducted in US
Egan et al. [14]	NR. Study conducted in US
Gram et al. [33]	NR, but study conducted in Norway and Sweden
Hirose et al. [69]	NR, but study conducted in Japan
La Vecchia et al. [15]	NR, but study conducted in Italy
Magnusson et al. [70]	NR, but study conducted in Sweden
Nomura et al. [19]	NR. Study conducted in US
Peplonska et al. [71]	NR, but study conducted in Poland
Swerdlow et al. [72]	NR. Study subjects from Denmark, England and Wales, Finland, and Sweden
Ursin et al. [73]	NR. Study conducted in US
Weiderpass et al. [74]	NR, but study conducted in Norway and Sweden
UK National Case–Control Study Group [75]	NR. Study conducted in UK
Colditz et al. [21]	NR. Study conducted in US
Harris et al. [76]	NR. Study conducted in US
Lipnick et al. [77]	NR. Study conducted in US
Olsson et al. [78]	NR, but study conducted in Sweden
Paul et al. [79]	Ethnic group. Cases: Non-Maori 829, Maori 62. Controls: Non-Maori: 1774, Maori 90. Study conducted in New Zealand
Tavani et al. [80]	NR, but study conducted in Italy
No author listed (Division of Reproductive Health, Centers for Disease Control) [81]	Race. Cases: White 81.7%, Black 11.8%, Other 6.5%. Controls: White 83.1%, Black 10.8%, Other 6.1%. Study conducted in US
Claus et al. [82]	Ethnicity. Cases: White 762 (87.1%), Black 57 (6.5%), Other 21 (2.4%), Missing 35 (4.0%). Controls: White 912 (91.3%), Black 54 (5.4%), Other 22 (2.2%), Missing 11 (1.1%). Study conducted in US
Rohan et al. [83]	NR. Study conducted in Australia
Lando et al. [84]	Race. White 85.4%, Black 13.8%, Other 0.8%. Study conducted in US
Nomura et al. [85]	Cases: Japanese 183, Caucasian 161. Same for hospital and neighborhood controls. Study conducted in US
Sellers et al. [86]	NR. Study conducted in US
White et al. [12]	Race/ethnicity. Low alcohol consumption: non-Hispanic white 17,181 (83.1%), other 3488 (16.9%). Medium alcohol consumption: non-Hispanic white 14,314 (89.1%), other 1744 (10.9%). High alcohol consumption: non-Hispanic white 4682 (91.1%), other 455 (8.9%). Study subjects from US or Puerto Rico
Brinton et al. [87]	Because most of the individuals participating in the BCDDP were white, the present analysis was restricted to the 405 cases of breast cancer detected among white women (91.4% of the total respondents) and to the 1,156 white controls. Study conducted in US Religion. Cases: 10.9% Jewish. Controls: 12.9% Jewish
Carpenter et al. [88]	NR. Study conducted in US
Cerhan et al. [89]	NR. Study conducted in US
Couch et al. [29]	The initial study was restricted to Caucasian women because very few minority women were available for meaningful analysis. Study conducted in US
Dinger et al. [90]	NR but study conducted in Germany
Grabrick et al. [91]	NR. Study conducted in US
Jones et al. [28]	NR. Study conducted in the UK
Katsouyanni et al. [17]	NR but study conducted in Greece
Kim et al. [18]	NR. Study conducted in US
Niehoff et al. [92]	Race/ethnicity. < 1 h per week of physical activity: non-Hispanic white 13,601 (79.1%), non-Hispanic black 1890 (11.0%), Hispanic 1197 (7.0%), other 503 (2.9%), missing 1. 1–6 h per week of physical activity: non-Hispanic white 24,897 (85.7%), non-Hispanic black 2284 (7.9%), Hispanic 1162 (4.0%), other 697 (2.4%), missing 6. ≥ 7 h per week of physical activity: non-Hispanic white 3892 (87.7%), non-Hispanic black 274 (6.2%), Hispanic 144 (3.3%), other 126 (2.8%), missing 2. Study conducted in US and Puerto Rico
Peters et al. [93]	Race/ethnicity. White 89.9%, Black 5.5%, Hispanic 1.9%, Asian/Pacific Islander/Native American 1.5%. Study conducted in US
Sellers et al. [94]	NR. Study conducted in US
Suzuki et al. [34]	NR but study conducted in Japan
Tehard et al. [95]	NR but study conducted in France
Vachon et al. [16]	NR. Study conducted in US
Verloop et al. [96]	NR but study conducted in the Netherlands
Brinton et al. [97]	Only used white study subjects. Study conducted in US
White et al. [98]	All cases and controls were white. Study conducted in US
Ravnihar et al. [99]	NR but study conducted in Slovenia
Pesch et al. [100]	All cases and controls were of Caucasian ethnicity. Study conducted in Germany
Huang et al. [13]	NR but study conducted in Japan

### Risk of bias

Risk of bias in the included studies was assessed based on the consistency of the measurement of exposure and outcome, the adequacy of accounting for confounding variables, and the sufficiency of follow-up.

Exposure was measured very inconsistently across studies of the same modifiable risk factors. Studies differed in whether the exposure was measured in the past, present, or specific periods of life, or based on duration as well as the level of exposure investigated within these time frames. A few studies did not specify exposure, in which case the exposure was classified as ever use. Tables 3, 4, 5, and 6 illustrate the heterogeneity of exposure definitions for smoking and alcohol. Despite the variability demonstrated, smoking and alcohol exposures are defined more consistently across studies than MHT/HC, BMI/weight, or physical activity exposures. Not only were studies of these modifiable risk factors more variable in the way exposures were defined but also more likely to undertake further subgroup divisions. For example, studies of BMI/weight and HC/MHT commonly, but not universally, assessed the impact of menopausal status or utilized postmenopausal (POM) or pre-menopausal (PRM) participants only.

There was less variation in the way studies measured and classified non-modifiable risk factors. Four studies including women with *BRCA* mutations included women with *BRCA1* mutations only. The remaining studies included women with *BRCA1* or *BRCA2* mutations and studied them combined or separately. Most studies of women with FHBC included women with a 1st-degree relative with BC. Nine studies included women with 1st- or 2nd-degree relatives with BC, one study included only women with a 2nd-degree relative, and six studies did not specify the degree of relatedness of the relative.

The outcome was reported largely consistently across studies as breast cancer. Though many studies did not specify the type of breast cancer (DCIS vs. LCIS vs. invasive) with which their participants were diagnosed, those studies that did, most commonly included participants with invasive breast carcinoma (IBC) and occasionally additionally included DCIS. Two studies used only participants with DCIS. Breast cancer status was collected by self- or proxy-report, physician report, or health records.

Most of the studies included adjusted for at least some of the other known risk factors such as age, age at menarche, menopausal status, age at first birth, parity, as well as those factors included in this review. In those studies that reported follow-up, it ranged from 4 to 26 years with an average of 9.9 years and a median of 7.8 years.

### Imprecision

There was significant heterogeneity in exposures measured as discussed above; thus, it would be very difficult to compare confidence intervals directly or to perform a statistical test of heterogeneity.

### Indirectness

Most of the studies included directly assessed the question of how modifiable risk factors affect women with non-modifiable risk of BC in the main analysis or as a subgroup analysis. However, small sample sizes in many of the articles likely reduce the generalizability of their findings. Sample sizes in the included studies ranged from 68 to 4,984 women with *BRCA* mutations and 24 to 43,713 women with FHBC, including both cases and controls. However, when studies investigated specific exposures, such as glasses/day of alcohol, sample sizes could fall as low as 4 participants.

### Publication bias

Although it would not be possible to assess publication bias using a funnel plot, it is generally assumed that unpublished studies are mostly small null studies.

### Race/ethnicity data

Studies varied in reporting of the information about the racial/ethnic distribution of the study populations used. While some detailed the demographics of participants by race (such as White, Black, etc.), others reported on the numbers of French-Canadian and Jewish participants. There were also a significant number of studies that did not provide any information on race or ethnicity for the study populations used. The studies included in this review represent a wide number of countries with several using international cohorts, though most were conducted in Western countries such as the USA, Canada, Europe, and Australia. Additionally, a few studies represented Asian countries including Japan and Korea. With the lack of detailed and consistent reporting of racial and ethnic demographics of study populations used, it is difficult to assess the generalizability of findings.

## Discussion

All women face some risk of breast cancer and modifiable risk factors have been demonstrated to be an important component of that risk [2]. Some risk factors such as low physical activity and alcohol use have shown a consistent pattern of increasing breast cancer risk [35]. Though trends in breast cancer risk related to other risk factors such as hormonal contraception use and body mass index have been linked to breast cancer risk, the relationship differs based on menopausal status and age. Given the smaller numbers of women with *BRCA* mutations or family history, necessitating further subdivision of subjects by age or menopausal status makes the elucidation of these trends more challenging.

Despite robust research on modifiable risk factors for breast cancer, it has remained unclear how, if at all, modifiable risk factors affect breast cancer risk in women with underlying high risk due to inherited non-modifiable risk factors. Particularly as women become more aware of both their family histories and commonly tested genetic polymorphisms, such as in *BRCA1/2*, information on modifiable risk factors for high-risk women will become even more critical. It is also important to consider how these modifiable factors affect the risk of other cancers that patients with *BRCA1/2* mutations are at increased risk of, such as ovarian cancer [36].

Although this review included ten or more studies with women with positive family history for every investigated modifiable risk factor, fewer were available for most modifiable risk factors in *BRCA1/2* mutation carriers, and only one study investigated women with a familial cancer syndrome, despite the search including several familial cancer syndromes. Furthermore, the studies in this review present risk estimates based on disparate exposures to modifiable risk factors and often suffer from low sample sizes and low quality.

The inconsistency in the classification and measurement of exposures to modifiable risk factors limited the direct comparison of studies and made it problematic to perform a meta-analysis as well as to draw conclusions on the high-priority modifiable risk factors for women at high risk of BC. Summarizing across all exposures for each modifiable risk factor, the data support that women at high risk of BC are similarly or less strongly affected by the commonly accepted modifiable risk factors for breast cancer. In studies on women with positive family history, very few studies indicated that physical activity or elevated pre-menopausal BMI/weight increased risk or that HC/MHT, smoking, postmenopausal elevated BMI/weight or alcohol use decreased risk, rather they were null or associated with increased risk. Studies of women with *BRCA1/2* mutations demonstrated similar though more varied trends with smaller numbers of studies.

The discrepancies in modifiable risk factor exposure also increased risk of bias, decreasing quality. Quality was adequate in other areas such as indirectness and participant classification; however, imprecision could not be assessed due to heterogeneity. The ability to compare studies would improve if the same definitions of exposures were utilized. Alternatively, it could also be worthwhile to obtain the original participant data and to apply consistent definitions retrospectively to accurately compare cohorts.

## Conclusion

With growing awareness of family history and utilization of direct-to-consumer genetic testing, it will become easier to conduct higher-powered studies on this subject. In order for future studies to be more meaningful to women considering lifestyle changes, standardization of the reporting of exposures would be beneficial. Although this review suggests that women with high risk could benefit from following the same guidelines on modifiable risk used for women without underlying risk, such as maintaining an active lifestyle with low levels of tobacco and alcohol use, due to the limitations and heterogeneity of existing literature, women at high risk of breast cancer would benefit from further investigations into these and other modifiable risk factors.

## Supplementary Information

**Additional file 1**. Contraception/Menopausal Hormone Therapy and Breast Cancer Risk in Women with BRCA Mutations (n=13/n=4) and Family History (n=32). **A** Demonstrates the relationship between use of hormonal contraception (HC) and BC risk in women with BRCA mutations. Each bar in the figure represents all of the included studies (n = total number of studies) that reported results on the specified HC exposure, separated by BRCA mutation, if provided. Each bar is divided based on the proportion of included studies that demonstrated an increased risk, decreased risk, or no association with risk of breast cancer due to the specified HC exposure. Within each HC exposure, each study is represented only once. However, because the category “all HC use” combines the results of all other exposure categories, studies may be represented more than once, if the results differ by exposure (e.g. increase risk with ever use of HC and no association with use before first full term pregnancy). Numbers on the “all HC use” bars indicate the range of risk estimates from studies when reported as a ratio measure (OR/RR/HR). Results from studies reporting only* p*-values or other measures that did not indicate magnitude of effect are not included in these ranges. About one third of the data on HC and BC risk indicated increased risk, a small proportion indicated decreased risk and the majority indicated no association with risk of BC. For formulation, all included studies relied on use before and after 1975 as a proxy because HC included higher doses of estrogen prior to 1975. Because all studies reporting on use included data for use before 1975, the exposure was defined in this way. However, one study also reported that use after 1975 increased risk in women with BRCA1 and combined BRCA1/2 mutations and had no association with risk in women with BRCA2 mutations. Another study reported that use after 1975 had no association with risk in women with combined BRCA1/2 mutations. Please see Additional file 4: Table S1 for all studies cited. **B** Demonstrates the relationship between use of menopausal hormone therapy (MHT) and BC risk in women with BRCA mutations. Each bar in the figure represents all of the included studies (n = total number of studies) that reported results on the specified MHT exposure, separated by BRCA mutation, if provided. Each bar is divided based on the proportion of included studies that demonstrated an increased risk, decreased risk, or no association with risk of breast cancer due to the specified MHT exposure. Within each MHT exposure, each study is represented only once. However, because the category “all MHT use” combines the results of all other exposure categories, studies may be represented more than once, if the results differ by exposure (e.g. decrease risk with ever use of MHT and no association with duration of MHT use). Numbers on the “all MHT use” bars indicate the range of risk estimates from studies when reported as a ratio measure (OR/RR/HR). Results from studies reporting only * p*-values or other measures that did not indicate magnitude of effect are not included in these ranges. Most of the data included on MHT and BC risk indicated no association and a small proportion demonstrated increased or decreased risk of BC. The two studies that evaluated MHT containing unopposed estrogen were included in the figure (formulation), these studies both additionally found that MHT with estrogen and progesterone had no association with risk of BC. Please see Additional file 4: Table S1 for all studies cited. **C** Demonstrates the relationship between use of menopausal hormone therapy (MHT) or hormonal contraception (HC) and BC risk in women with family history of BC (FHBC). Each bar in the figure represents all of the included studies (n = total number of studies) that reported results on the specified exposure, separated into MHT and HC. Each bar is divided based on the proportion of included studies that demonstrated an increased risk, decreased risk, or no association with risk of breast cancer due to the specified MHT exposure. Within each MHT or HC exposure, each study is represented only once. However, because the category “all MHT/HC use” combines the results of all other exposure categories, studies may be represented more than once, if the results differ by exposure (e.g. increase risk with ever use of HC and no association with duration of HC use). Numbers on the “all MHT use” bars indicate the range of risk estimates from studies when reported as a ratio measure (OR/RR/HR). Results from studies reporting only * p*-values or other measures that did not indicate magnitude of effect are not included in these ranges. Most of the included data indicated no association between MHT or HC exposures and BC risk and a small proportion demonstrated increased or decreased risk of BC. Additional exposures reported in only a single study and thus not included in the figure were HC taken before 1975 (higher dose), which showed increased risk and use of estrogen only or combined estrogen and progesterone MHT, which demonstrated no difference in risk. Finally, one study evaluated duration of MHT use during menopause as well as in women greater than 50 years old. Only MHT use during menopause was significant and was included in the figure. Please see Additional file 4: Table S1 for all studies cited.

**Additional file 2**. BMI/Weight and Breast Cancer Risk in Women with BRCA Mutations (n=8) and Family History (n=12). **A** Demonstrates the relationship between body mass index (BMI) or weight and BC risk in women with BRCA mutations. Each bar in the figure represents all of the included studies (n = total number of studies) that reported results on the specified measure of BMI or weight, separated by effect on post-menopausal (POM) or pre-menopausal (PRM) BC, if provided. Each bar is divided based on the proportion of included studies that demonstrated an increased risk, decreased risk, or no association with risk of BC due to the specified BMI or weight measure. Within each BMI or weight category, each study is represented only once. However, because the category “any elevated weight/BMI” combines the results of all other exposure categories, studies may be represented more than once, if the results differ by exposure (e.g. increase risk with current weight and no association with adolescent BMI). Numbers on the “any elevated weight/BMI” bars indicate the range of risk estimates from studies when reported as a ratio measure (OR/RR/HR). Results from studies reporting only * p*-values or other measures that did not indicate magnitude of effect are not included in these ranges. Approximately half of the data included on BMI or weight and BC risk indicated no association with BC risk and about half demonstrated increased or decreased risk of BC. Because most studies looked at high or gain of BMI or weight, studies reporting on low or loss of BMI or weight were not included in the figure. One study found that low current BMI increased risk of PRM BC in women with BRCA2 mutations and had no association with risk of PRM BC in women with BRCA1 mutations. Another study found that low current weight had no association with risk of PRM BC and another found that weight loss decreased risk of BC in women with BRCA1 and BRCA1/2 combined but had no association with BC in women with BRCA2 mutations. No other studies had different results for BRCA1, BRCA2, or combined BRCA1/2 mutation carriers. Please see Additional file 4: Table S1 for all studies cited. **B** Demonstrates the relationship between body mass index (BMI) or weight and BC in women with FHBC. Each bar in the figure represents all included studies (n = total number of studies) that reported results on BMI or weight, separated by risk of post-menopausal (POM) or pre-menopausal (PRM) BC. Each bar is divided based on the proportion of included studies that demonstrated increased, decreased, or no association with risk of BC. Within each BMI or weight category, each study is represented only once. However, because the category “any elevated weight/BMI” combines the results of all other exposure categories, studies may be represented more than once, if the results differ by exposure (e.g. increase risk with current weight and no association with adolescent BMI). Numbers on the “any elevated weight/BMI” bars indicate the range of risk estimates from studies when reported as a ratio measure (OR/RR/HR). Results from studies reporting only * p*-values or other measures that did not indicate magnitude of effect are not included in these ranges. Half of the data included on the effect of BMI or weight on POM BC demonstrated increased risk and half reported no association or decreased risk. All studies of the effect of BMI/weight on PRM BC reported no association. The only study that did not separate POM and PRM BC found that adolescent BMI had no association with risk of BC and adolescent weight increased risk of BC. This study additionally assessed low weight at age 12 years old, which showed no association and weight at age 18 years old, which showed no association with BC. Because most studies looked at high BMI/weight, if studies reported low BMI/weight, those data were not included in the figure. One study found no association between low adolescent BMI and BC. Additional exposures reported in a single study and thus not included in the figure were somatotype at 7 years old, which was not associated with BC risk and waist-to-hip ratio, which was associated with increased risk of POM BC. Please see Additional file 4: Table S1 for all studies cited.

**Additional file 3**. Physical Activity and Breast Cancer Risk in Women with BRCA Mutations (n=5) and Family History (n=15). **A** Demonstrates the relationship between physical activity and BC risk in women with BRCA mutations. Each bar in the figure represents all of the included studies (n = total number of studies) that reported results on the specified measure of physical activity. Each bar is divided based on the proportion of included studies that demonstrated an increased risk, decreased risk, or no association with risk of BC due to the specified physical activity measure. Within each physical activity category, each study is represented only once. However, because the category “all physical activity” combines the results of all other exposure categories, studies may be represented more than once, if the results differ by exposure (e.g. decrease risk with adult physical activity and no association with lifetime physical activity). Numbers on the “all physical activity” bars indicate the range of risk estimates from studies when reported as a ratio measure (OR/RR/HR). Results from studies reporting only * p*-values or other measures that did not indicate magnitude of effect are not included in these ranges. Most of the data on physical activity indicated no association and about a third demonstrated decreased risk of BC. Additional alcohol exposures that appeared in only one article and thus were not presented in this figure include hours/week, duration, sports activity during adolescence and intensity of activity during adolescence, which all showed no association with BC risk as well as current physical activity, which had no association with BC risk in women with BRCA1 mutations and decreased risk in women with BRCA2 mutations. No other studies had different results for BRCA1 and BRCA2 mutation carriers. Please see Additional file 4: Table S1 for all studies cited. **B** Demonstrates the relationship between physical activity and BC risk in women with FHBC. Each bar in the figure represents all of the included studies (n = total number of studies) that reported results on the specified measure of physical activity. Each bar is divided based on the proportion of included studies that demonstrated an increased risk, decreased risk, or no association with risk of BC due to the specified physical activity measure. Within each physical activity category, each study is represented only once. However, because the category “all physical activity” combines the results of all other exposure categories, studies may be represented more than once, if the results differ by exposure (e.g. decrease risk with hours/week and no association with intensity). Numbers on the “all physical activity” bars indicate the range of risk estimates from studies when reported as a ratio measure (OR/RR/HR). Results from studies reporting only * p*-values or other measures that did not indicate magnitude of effect are not included in these ranges. Over half the data included indicated no association between physical activity and BC risk and a little less than half indicated decreased risk of BC. Additional exposures reported in only a single study and thus not included in the figure were adolescent physical activity and physical activity from 22 years old to menopause, which both demonstrated increased risk; physical activity post-menopause, which was not associated with BC risk; and minutes per session, which showed a decrease in risk of BC. Studies reporting on current physical activity cited many different types of current activity, such as outdoor and occupational activities, light household work, and less than 80% sedentary activities. Finally, one study with data on hours/week of physical activity reported on hours/week of vigorous and hours/week of moderate intensity physical activity. Hours/week of vigorous activity demonstrated decreased risk of BC and was included in the figure, whereas, hours/week of moderate activity had no association with BC risk and was not included in the figure. Please see Additional file 4: Table S1 for all studies cited.

**Additional file 4**. Characteristics of included prospective studies on family history of breast cancer.

**Additional file 5**. Characteristics of included retrospective studies on family history of breast cancer.

**Additional file 6**. Full search strategy.

## Data Availability

Data sharing is not applicable to this article as no datasets were generated or analyzed during the current study.

## Abbreviations

BC: Breast cancer
FHBC: Family history of breast cancer
HC: Hormonal contraception
MHT: Menopausal hormone therapy
BMI: Body Mass Index
DC: Ductal carcinoma
LC: Lobular carcinoma
DCIS: Ductal carcinoma in situ
LCIS: Lobular carcinoma in situ
IBC: Invasive breast cancer
POM: Post-menopausal
PRM: Pre-menopausal
PRISMA: Preferred Reporting Items for Systematic Reviews and Meta-Analyses
GRADE: Grading of Recommendations, Assessment, Development and Evaluation
CI: Confidence interval
