# Supplementary material for: Modifiable risk factors in women at high risk of breast cancer: a systematic review
Source: Breast Cancer Res. 2023 Apr 24;25:45. doi: 10.1186/s13058-023-01636-1 (PMC10123992; doi:10.1186/s13058-023-01636-1)
Supplement: Supplementary file 1 — Additional file 1. Contraception/Menopausal Hormone Therapy and Breast Cancer Risk in Women with BRCA Mutations (n=13/n=4) and Family History (n=32). A Demonstrates the relationship between use of hormonal contraception (HC) and BC risk in women with BRCA mutations. Each bar in the figure represents all of the included studies (n = total number of studies) that reported results on the specified HC exposure, separated by BRCA mutation, if provided. Each bar is divided based on the proportion of included studies that demonstrated an increased risk, decreased risk, or no association with risk of breast cancer due to the specified HC exposure. Within each HC exposure, each study is represented only once. However, because the category “all HC use” combines the results of all other exposure categories, studies may be represented more than once, if the results differ by exposure (e.g. increase risk with ever use of HC and no association with use before first full term pregnancy). Numbers on the “all HC use” bars indicate the range of risk estimates from studies when reported as a ratio measure (OR/RR/HR). Results from studies reporting only p-values or other measures that did not indicate magnitude of effect are not included in these ranges. About one third of the data on HC and BC risk indicated increased risk, a small proportion indicated decreased risk and the majority indicated no association with risk of BC. For formulation, all included studies relied on use before and after 1975 as a proxy because HC included higher doses of estrogen prior to 1975. Because all studies reporting on use included data for use before 1975, the exposure was defined in this way. However, one study also reported that use after 1975 increased risk in women with BRCA1 and combined BRCA1/2 mutations and had no association with risk in women with BRCA2 mutations. Another study reported that use after 1975 had no association with risk in women with combined BRCA1/2 mutations. Please see Additio [file 13058_2023_1636_MOESM1_ESM.docx]

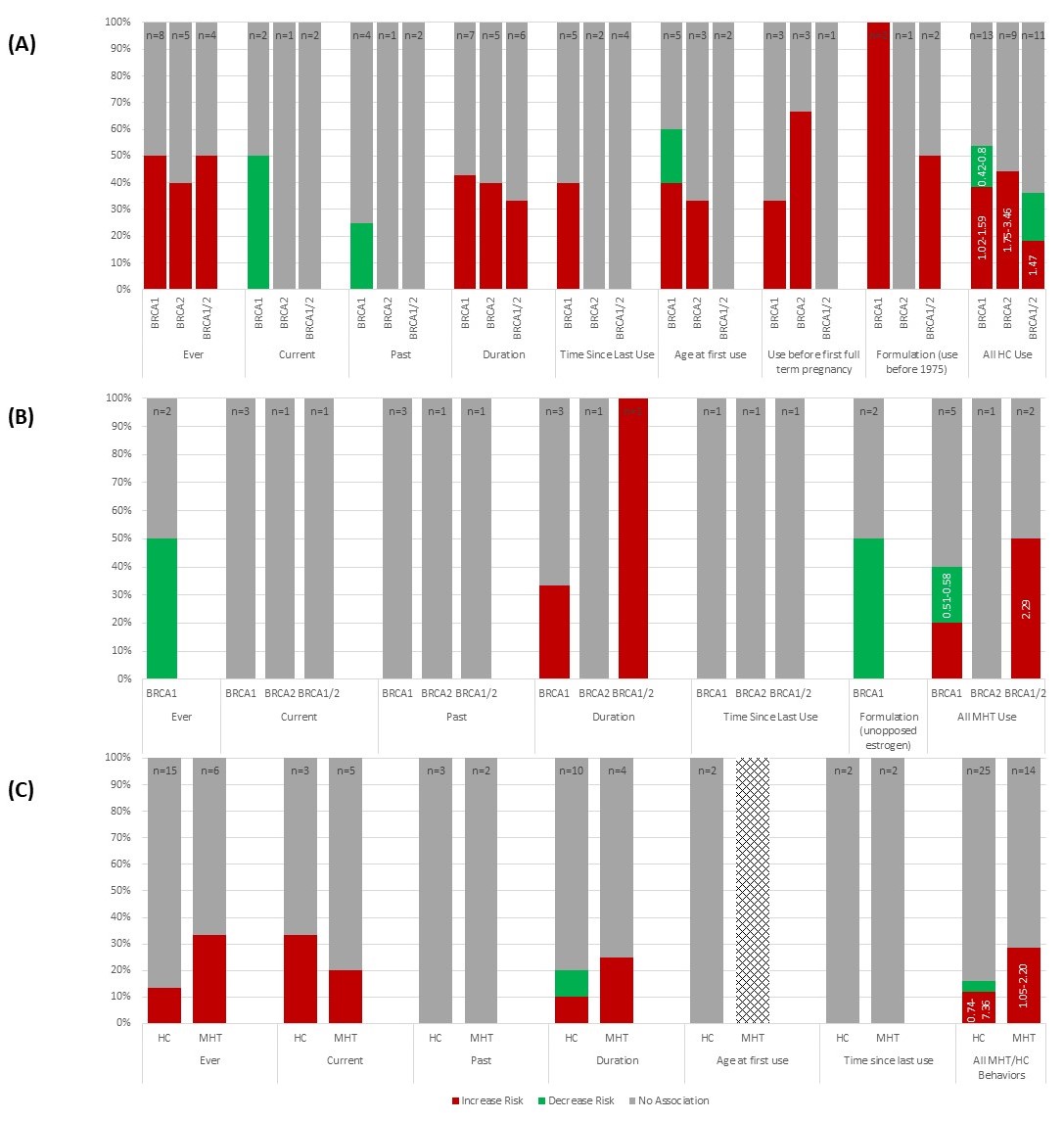
**Additional Figure 1: Hormonal Contraception/Menopausal Hormone Therapy and Breast Cancer Risk in Women with *BRCA* Mutations (n=13/n=4) and Family History (n=32)**

**Additional Figure 1A** demonstrates the relationship between use of hormonal contraception (HC) and BC risk in women with *BRCA* mutations. Each bar in the figure represents all of the included studies (n = total number of studies) that reported results on the specified HC exposure, separated by *BRCA* mutation, if provided. Each bar is divided based on the proportion of included studies that demonstrated an increased risk, decreased risk, or no association with risk of breast cancer due to the specified HC exposure. Within each HC exposure, each study is represented only once. However, because the category “all HC use” combines the results of all other exposure categories, studies may be represented more than once, if the results differ by exposure (e.g. increase risk with ever use of HC and no association with use before first full term pregnancy). Numbers on the “all HC use” bars indicate the range of risk estimates from studies when reported as a ratio measure (OR/RR/HR). Results from studies reporting only p-values or other measures that did not indicate magnitude of effect are not included in these ranges.

About one third of the data on HC and BC risk indicated increased risk, a small proportion indicated decreased risk and the majority indicated no association with risk of BC. For formulation, all included studies relied on use before and after 1975 as a proxy because HC included higher doses of estrogen prior to 1975. Because all studies reporting on use included data for use before 1975, the exposure was defined in this way. However, one study also reported that use after 1975 increased risk in women with *BRCA*1 and combined *BRCA1/2* mutations and had no association with risk in women with BRCA2 mutations. Another study reported that use after 1975 had no association with risk in women with combined *BRCA1/2* mutations. Please see Table 1 for all studies cited.

**Additional Figure 1B** demonstrates the relationship between use of menopausal hormone therapy (MHT) and BC risk in women with *BRCA* mutations. Each bar in the figure represents all of the included studies (n = total number of studies) that reported results on the specified MHT exposure, separated by *BRCA* mutation, if provided. Each bar is divided based on the proportion of included studies that demonstrated an increased risk, decreased risk, or no association with risk of breast cancer due to the specified MHT exposure. Within each MHT exposure, each study is represented only once. However, because the category “all MHT use” combines the results of all other exposure categories, studies may be represented more than once, if the results differ by exposure (e.g. decrease risk with ever use of MHT and no association with duration of MHT use). Numbers on the “all MHT use” bars indicate the range of risk estimates from studies when reported as a ratio measure (OR/RR/HR). Results from studies reporting only p-values or other measures that did not indicate magnitude of effect are not included in these ranges.

Most of the data included on MHT and BC risk indicated no association and a small proportion demonstrated increased or decreased risk of BC. The two studies that evaluated MHT containing unopposed estrogen were included in the figure (formulation), these studies both additionally found that MHT with estrogen and progesterone had no association with risk of BC. Please see Table 1 for all studies cited.

**Additional Figure 1C** demonstrates the relationship between use of menopausal hormone therapy (MHT) or hormonal contraception (HC) and BC risk in women with family history of BC (FHBC). Each bar in the figure represents all of the included studies (n = total number of studies) that reported results on the specified exposure, separated into MHT and HC. Each bar is divided based on the proportion of included studies that demonstrated an increased risk, decreased risk, or no association with risk of breast cancer due to the specified MHT exposure. Within each MHT or HC exposure, each study is represented only once. However, because the category “all MHT/HC use” combines the results of all other exposure categories, studies may be represented more than once, if the results differ by exposure (e.g. increase risk with ever use of HC and no association with duration of HC use). Numbers on the “all MHT use” bars indicate the range of risk estimates from studies when reported as a ratio measure (OR/RR/HR). Results from studies reporting only p-values or other measures that did not indicate magnitude of effect are not included in these ranges.

Most of the included data indicated no association between MHT or HC exposures and BC risk and a small proportion demonstrated increased or decreased risk of BC. Additional exposures reported in only a single study and thus not included in the figure were HC taken before 1975 (higher dose), which showed increased risk and use of estrogen only or combined estrogen and progesterone MHT, which demonstrated no difference in risk. Finally, one study evaluated duration of MHT use during menopause as well as in women greater than 50 years old. Only MHT use during menopause was significant and was included in the figure. Please see Additional Table 1 for all studies cited.
