# Supplementary material for: Modifiable risk factors in women at high risk of breast cancer: a systematic review
Source: Breast Cancer Res. 2023 Apr 24;25:45. doi: 10.1186/s13058-023-01636-1 (PMC10123992; doi:10.1186/s13058-023-01636-1)
Supplement: Supplementary file 2 — Additional file 2. BMI/Weight and Breast Cancer Risk in Women with BRCA Mutations (n=8) and Family History (n=12). A Demonstrates the relationship between body mass index (BMI) or weight and BC risk in women with BRCA mutations. Each bar in the figure represents all of the included studies (n = total number of studies) that reported results on the specified measure of BMI or weight, separated by effect on post-menopausal (POM) or pre-menopausal (PRM) BC, if provided. Each bar is divided based on the proportion of included studies that demonstrated an increased risk, decreased risk, or no association with risk of BC due to the specified BMI or weight measure. Within each BMI or weight category, each study is represented only once. However, because the category “any elevated weight/BMI” combines the results of all other exposure categories, studies may be represented more than once, if the results differ by exposure (e.g. increase risk with current weight and no association with adolescent BMI). Numbers on the “any elevated weight/BMI” bars indicate the range of risk estimates from studies when reported as a ratio measure (OR/RR/HR). Results from studies reporting only p-values or other measures that did not indicate magnitude of effect are not included in these ranges. Approximately half of the data included on BMI or weight and BC risk indicated no association with BC risk and about half demonstrated increased or decreased risk of BC. Because most studies looked at high or gain of BMI or weight, studies reporting on low or loss of BMI or weight were not included in the figure. One study found that low current BMI increased risk of PRM BC in women with BRCA2 mutations and had no association with risk of PRM BC in women with BRCA1 mutations. Another study found that low current weight had no association with risk of PRM BC and another found that weight loss decreased risk of BC in women with BRCA1 and BRCA1/2 combined but had no association with BC in women with BRCA2 [file 13058_2023_1636_MOESM2_ESM.docx]

**
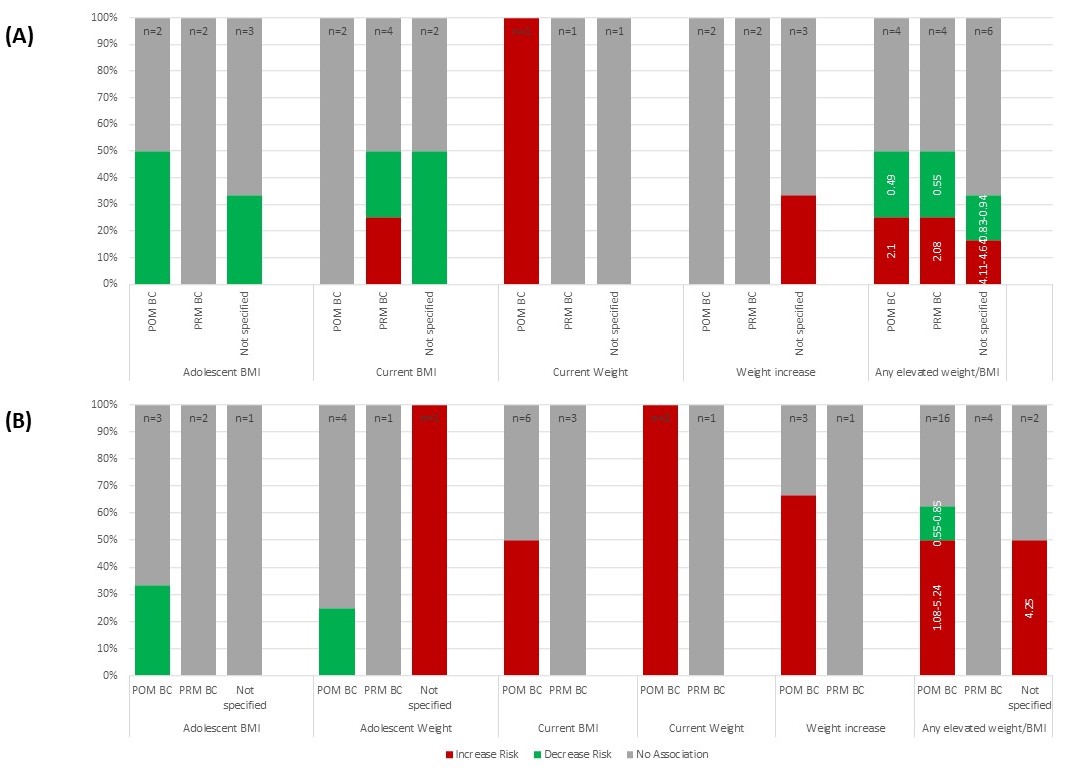
**

**Additional Figure 2: BMI/Weight and Breast Cancer Risk in Women with *BRCA* Mutations (n=8) and Family History (n=12)**

**Additional Figure 2A** demonstrates the relationship between body mass index (BMI) or weight and BC risk in women with *BRCA* mutations. Each bar in the figure represents all of the included studies (n = total number of studies) that reported results on the specified measure of BMI or weight, separated by effect on post-menopausal (POM) or pre-menopausal (PRM) BC, if provided. Each bar is divided based on the proportion of included studies that demonstrated an increased risk, decreased risk, or no association with risk of BC due to the specified BMI or weight measure. Within each BMI or weight category, each study is represented only once. However, because the category “any elevated weight/BMI” combines the results of all other exposure categories, studies may be represented more than once, if the results differ by exposure (e.g. increase risk with current weight and no association with adolescent BMI). Numbers on the “any elevated weight/BMI” bars indicate the range of risk estimates from studies when reported as a ratio measure (OR/RR/HR). Results from studies reporting only p-values or other measures that did not indicate magnitude of effect are not included in these ranges.

Approximately half of the data included on BMI or weight and BC risk indicated no association with BC risk and about half demonstrated increased or decreased risk of BC. Because most studies looked at high or gain of BMI or weight, studies reporting on low or loss of BMI or weight were not included in the figure. One study found that low current BMI increased risk of PRM BC in women with *BRCA2* mutations and had no association with risk of PRM BC in women with *BRCA1* mutations. Another study found that low current weight had no association with risk of PRM BC and another found that weight loss decreased risk of BC in women with *BRCA1* and *BRCA1/2* combined but had no association with BC in women with *BRCA2* mutations. No other studies had different results for *BRCA1*, *BRCA2*, or combined *BRCA1/2* mutation carriers. Please see Table 1 for all studies cited.

**Additional Figure 2B** demonstrates the relationship between body mass index (BMI) or weight and BC in women with FHBC. Each bar in the figure represents all included studies (n = total number of studies) that reported results on BMI or weight, separated by risk of post-menopausal (POM) or pre-menopausal (PRM) BC. Each bar is divided based on the proportion of included studies that demonstrated increased, decreased, or no association with risk of BC. Within each BMI or weight category, each study is represented only once. However, because the category “any elevated weight/BMI” combines the results of all other exposure categories, studies may be represented more than once, if the results differ by exposure (e.g. increase risk with current weight and no association with adolescent BMI). Numbers on the “any elevated weight/BMI” bars indicate the range of risk estimates from studies when reported as a ratio measure (OR/RR/HR). Results from studies reporting only p-values or other measures that did not indicate magnitude of effect are not included in these ranges.

Half of the data included on the effect of BMI or weight on POM BC demonstrated increased risk and half reported no association or decreased risk. All studies of the effect of BMI/weight on PRM BC reported no association. The only study that did not separate POM and PRM BC found that adolescent BMI had no association with risk of BC and adolescent weight increased risk of BC. This study additionally assessed low weight at age 12 years old, which showed no association and weight at age 18 years old, which showed no association with BC. Because most studies looked at high BMI/weight, if studies reported low BMI/weight, those data were not included in the figure. One study found no association between low adolescent BMI and BC. Additional exposures reported in a single study and thus not included in the figure were somatotype at 7 years old, which was not associated with BC risk and waist-to-hip ratio, which was associated with increased risk of POM BC. Please see Additional Table 1 for all studies cited.
