# Supplementary material for: Modifiable risk factors in women at high risk of breast cancer: a systematic review
Source: Breast Cancer Res. 2023 Apr 24;25:45. doi: 10.1186/s13058-023-01636-1 (PMC10123992; doi:10.1186/s13058-023-01636-1)
Supplement: Supplementary file 3 — Additional file 3. Physical Activity and Breast Cancer Risk in Women with BRCA Mutations (n=5) and Family History (n=15). A Demonstrates the relationship between physical activity and BC risk in women with BRCA mutations. Each bar in the figure represents all of the included studies (n = total number of studies) that reported results on the specified measure of physical activity. Each bar is divided based on the proportion of included studies that demonstrated an increased risk, decreased risk, or no association with risk of BC due to the specified physical activity measure. Within each physical activity category, each study is represented only once. However, because the category “all physical activity” combines the results of all other exposure categories, studies may be represented more than once, if the results differ by exposure (e.g. decrease risk with adult physical activity and no association with lifetime physical activity). Numbers on the “all physical activity” bars indicate the range of risk estimates from studies when reported as a ratio measure (OR/RR/HR). Results from studies reporting only p-values or other measures that did not indicate magnitude of effect are not included in these ranges. Most of the data on physical activity indicated no association and about a third demonstrated decreased risk of BC. Additional alcohol exposures that appeared in only one article and thus were not presented in this figure include hours/week, duration, sports activity during adolescence and intensity of activity during adolescence, which all showed no association with BC risk as well as current physical activity, which had no association with BC risk in women with BRCA1 mutations and decreased risk in women with BRCA2 mutations. No other studies had different results for BRCA1 and BRCA2 mutation carriers. Please see Additional file 4: Table S1 for all studies cited. B Demonstrates the relationship between physical activity and BC risk in women with FHBC. Each bar in [file 13058_2023_1636_MOESM3_ESM.docx]

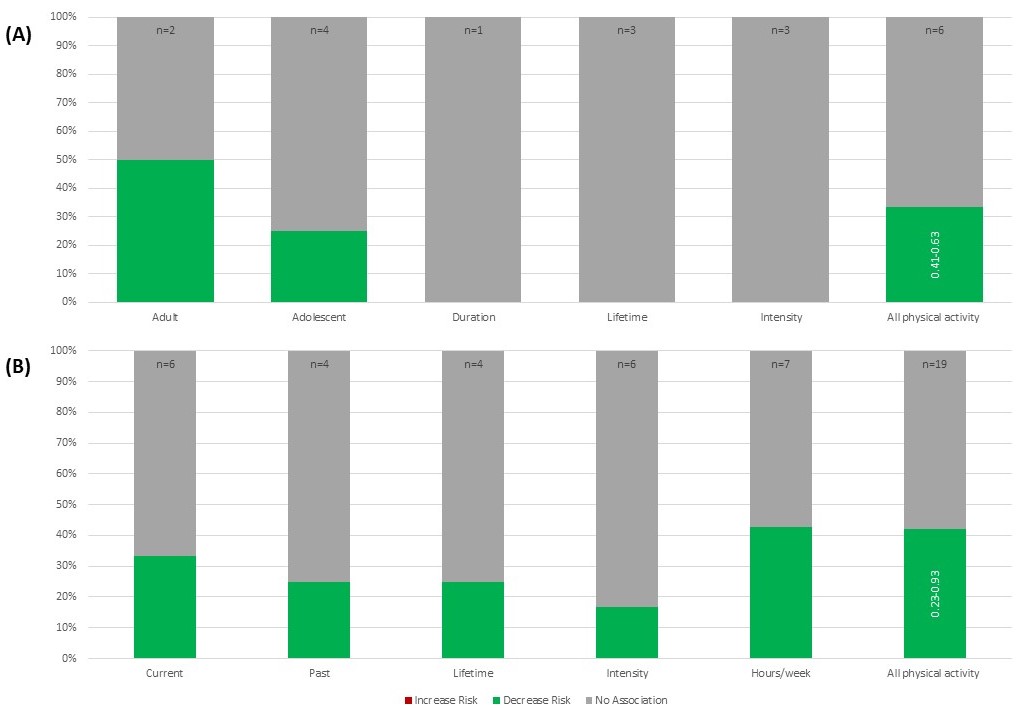


**Additional Figure 3: Physical Activity and Breast Cancer Risk in Women with *BRCA* Mutations (n=5) and Family History (n=15)**

**Additional Figure 3A** demonstrates the relationship between physical activity and BC risk in women with *BRCA* mutations. Each bar in the figure represents all of the included studies (n = total number of studies) that reported results on the specified measure of physical activity. Each bar is divided based on the proportion of included studies that demonstrated an increased risk, decreased risk, or no association with risk of BC due to the specified physical activity measure. Within each physical activity category, each study is represented only once. However, because the category “all physical activity” combines the results of all other exposure categories, studies may be represented more than once, if the results differ by exposure (e.g. decrease risk with adult physical activity and no association with lifetime physical activity). Numbers on the “all physical activity” bars indicate the range of risk estimates from studies when reported as a ratio measure (OR/RR/HR). Results from studies reporting only p-values or other measures that did not indicate magnitude of effect are not included in these ranges.

Most of the data on physical activity indicated no association and about a third demonstrated decreased risk of BC. Additional alcohol exposures that appeared in only one article and thus were not presented in this figure include hours/week, duration, sports activity during adolescence and intensity of activity during adolescence, which all showed no association with BC risk as well as current physical activity, which had no association with BC risk in women with *BRCA1* mutations and decreased risk in women with *BRCA2* mutations. No other studies had different results for *BRCA1* and *BRCA2* mutation carriers. Please see Table 1 for all studies cited.

**Additional Figure 3B** demonstrates the relationship between physical activity and BC risk in women with FHBC. Each bar in the figure represents all of the included studies (n = total number of studies) that reported results on the specified measure of physical activity. Each bar is divided based on the proportion of included studies that demonstrated an increased risk, decreased risk, or no association with risk of BC due to the specified physical activity measure. Within each physical activity category, each study is represented only once. However, because the category “all physical activity” combines the results of all other exposure categories, studies may be represented more than once, if the results differ by exposure (e.g. decrease risk with hours/week and no association with intensity). Numbers on the “all physical activity” bars indicate the range of risk estimates from studies when reported as a ratio measure (OR/RR/HR). Results from studies reporting only p-values or other measures that did not indicate magnitude of effect are not included in these ranges.

Over half the data included indicated no association between physical activity and BC risk and a little less than half indicated decreased risk of BC. Additional exposures reported in only a single study and thus not included in the figure were adolescent physical activity and physical activity from 22 years old to menopause, which both demonstrated increased risk; physical activity post-menopause, which was not associated with BC risk; and minutes per session, which showed a decrease in risk of BC. Studies reporting on current physical activity cited many different types of current activity, such as outdoor and occupational activities, light household work, and less than 80% sedentary activities. Finally, one study with data on hours/week of physical activity reported on hours/week of vigorous and hours/week of moderate intensity physical activity. Hours/week of vigorous activity demonstrated decreased risk of BC and was included in the figure, whereas, hours/week of moderate activity had no association with BC risk and was not included in the figure. Please see Additional Table 1 for all studies cited.
