# Supplementary material for: Modifiable risk factors in women at high risk of breast cancer: a systematic review
Source: Breast Cancer Res. 2023 Apr 24;25:45. doi: 10.1186/s13058-023-01636-1 (PMC10123992; doi:10.1186/s13058-023-01636-1)
Supplement: Supplementary file 4 — Additional file 4. Characteristics of included prospective studies on family history of breast cancer. [file 13058_2023_1636_MOESM4_ESM.docx]

| ADDITIONAL TABLE 1: CHARACTERISTICS OF INCLUDED PROSPECTIVE STUDIES ON FAMILY HISTORY OF BREAST CANCER | | | | | | | |
| --- | --- | --- | --- | --- | --- | --- | --- |
| Author | Sample Size | Alcohol | Smoking | MHT/hc | Bmi/weight | Physical activity | Notes |
| Nyante et al. (2014)^1^ | *186150 (7481 cases)* |  | ✓ |  |  |  | Number of women with family history not specified. All cases were prospectively ascertained. |
| Reynolds et al. (2004)^2^ | 13684 (381 cases) |  | ✓ |  |  |  | All cases were prospectively ascertained |
| Silvera et al. (2005)^3^ | 10914 (795 cases) |  |  | ✓-  0.74 (0.55-0.99)  (HC, duration) |  |  | All cases were prospectively ascertained |
| Bardia et al. (2008)^4^ | 4882 (437 cases) |  |  |  | ✓-  0.85 (0.74-0.98)  (high, 12y, POM) |  | All cases were prospectively ascertained |
| Colditz et al. (2012)^5^ | 10717 (686 cases) | ✓ |  | ✓+ | ✓ |  | All cases were prospectively ascertained |
| Gram et al. (2005)^6^ | *102,098 (1240 cases)* |  | ✓ |  |  |  | Number of women with family history not specified. All cases were prospectively ascertained. |
| Nomura et al. (2016)^7^ | 4755 (523 cases) | ✓ |  |  | ✓ | ✓ | All cases were prospectively ascertained |
| Weiderpass et al. (2004)^8^ | 4656 (75 cases) |  |  |  | ✓ |  | Article says 72 cases, numbers add up to 75. All cases were prospectively ascertained. |
| Colditz et al. (1996)^9^ | 5348 (311 cases) | ✓ |  | ✓ |  |  | All cases were prospectively ascertained |
| Lipnick et al. (1986)^10^ | 6740 (71 cases) |  |  | ✓ |  |  | All cases were prospectively ascertained |
| Olsson et al. (2001)^11^ | *28363 (434 cases)* |  |  | ✓+  2.20 (1.06-4.05)  (MHT, ever) |  |  | Number of women with family history not specified. All cases were prospectively ascertained. |
| Lando et al. (1999)^12^ | 542 |  |  | ✓ |  |  | Number of cases with family history not specified, but there were a total of 219. All cases were prospectively ascertained. |
| Sellers et al. (1997)^13^ | 4310 (170 cases) |  |  | ✓ |  |  | All cases were prospectively ascertained |
| White et al. (2017)^14^ | 41870 (1843 cases) | ✓+  1.35 (1.15-1.58)  (lifetime) |  |  |  |  | All cases were prospectively ascertained |
| Jones et al. (2017)^15^ | 15897 (457 cases) |  | ✓+  1.35 (1.12-1.62)  (ever) |  |  |  | All cases were prospectively ascertained |
| Kim et al. (2017)^16^ | 5722 (518 cases) | ✓+  1.33 (0.97-1.82)  (amount/day) |  |  |  |  | All cases were prospectively ascertained |
| Niehoff et al. (2019)^17^ | 50884 (3023 cases) |  |  |  |  | ✓-  0.89 (0.81–0.98)  (current)  0.85 (0.74-0.98)  (hrs/week) | All cases were prospectively ascertained |
| Peters et al. (2009)^18^ | 22674 (1193 cases) |  |  |  |  | ✓-  0.75 (0.62-0.91)  (hrs/week) | All cases were prospectively ascertained |
| Sellers et al. (2002)^19^ | 4564 (282 cases) |  |  |  | ✓-  0.55 (0.37-0.81)  (adolescent) |  | All cases were prospectively ascertained |
| Tehard et al. (2006)^20^ | 10373 (744 cases) |  |  |  |  | ✓-  0.62 (0.49-0.78)  (hrs/week) | All cases were prospectively ascertained. |
| Gong et al. (2016)^21^* | 1663 (502 cases) |  |  |  |  | ✓ |  |
| Cerhan et al. (2004)^22^* | 308 (31 cases) |  |  |  | ✓+  4.25 (1.71-10.5)  (12y) |  |  |
| Couch et al. (2001)^23^* | 138 (26 cases) |  | ✓+  1.8 (1.2-2.7)  (ever) |  |  |  |  |
| Grabrick et al. (2000)^24^* | 394 (38 cases) |  |  | ✓+  4.6 (2.0-10.7)  (HC, 1^st^ degree, ever)  3.3 (1.5-7.2)  (HC, 1^st^ degree, formulation) |  |  |  |
| Vachon et al. (2001)^25^* | 2067 (128 cases) | ✓+  2.45 (1.20-5.02)  (daily, 1^st^ degree) |  |  |  |  |  |
| *Secondary analysis of prospectively collected data  ✓Study presented data on the association of the modifiable risk factor with breast cancer. Association presented was positive (+), negative (-), or not significant (no +/- listed). Associations could be both (+) and (–) if multiple associations were presented.  Significant risk estimates (RR/OR/HR (95% CI)) from studies are listed. Results from studies reporting only p-values or other measures that did not indicate magnitude of effect are not included in this table. | | | | | | | |

**References**

1. Nyante SJ, Gierach GL, Dallal CM, et al. Cigarette smoking and postmenopausal breast cancer risk in a prospective cohort. *British Journal of Cancer* 2014;110(9):2339-47. doi: 10.1038/bjc.2014.132

2. Reynolds P, Hurley S, Goldberg DE, et al. Active smoking household passive smoking, and breast cancer: Evidence from the California Teachers Study. *Journal of the National Cancer Institute* 2004;96(1):29-37. doi: 10.1093/jnci/djh002

3. Silvera SAN, Miller AB, Rohan TE. Oral contraceptive use and risk of breast cancer among women with a family history of breast cancer: a prospective cohort study. *Cancer causes & control : CCC* 2005;16(9):1059-63.

4. Bardia A, Vachon CM, Olson JE, et al. Relative weight at age 12 and risk of postmenopausal breast cancer. *Cancer Epidemiology Biomarkers and Prevention* 2008;17(2):374-78. doi: 10.1158/1055-9965.EPI-07-0389

5. Colditz GA, Kaphingst KA, Hankinson SE, et al. Family history and risk of breast cancer: Nurses' health study. *Breast Cancer Research and Treatment* 2012;133(3):1097-104. doi: 10.1007/s10549-012-1985-9

6. Gram IT, Braaten T, Terry PD, et al. Breast cancer risk among women who start smoking as teenagers. *Cancer Epidemiology Biomarkers and Prevention* 2005;14(1):61-66.

7. Nomura SJO, Inoue-Choi M, Lazovich D, et al. WCRF/AICR recommendation adherence and breast cancer incidence among postmenopausal women with and without non-modifiable risk factors. *International Journal of Cancer* 2016;138(11):2602-15. doi: 10.1002/ijc.29994

8. Weiderpass E, Braaten T, Magnusson C, et al. A prospective study of body size in different periods of life and risk of premenopausal breast cancer. *Cancer Epidemiology Biomarkers and Prevention* 2004;13(7):1121-27.

9. Colditz GA, Rosner BA, Speizer FE. Risk factors for breast cancer according to family history of breast cancer. *Journal of the National Cancer Institute* 1996;88(6):365-71. doi: 10.1093/jnci/88.6.365

10. Lipnick RJ, Buring JE, Hennekens CH, et al. Oral contraceptives and breast cancer. A prospective cohort study. *JAMA* 1986;255(1):58-61.

11. Olsson H, Bladstrom A, Ingvar C, et al. A population-based cohort study of MHT use and breast cancer in southern Sweden. *British journal of cancer* 2001;85(5):674-7.

12. Lando JF, Heck KE, Brett KM. Hormone replacement therapy and breast cancer risk in a nationally representative cohort. *American Journal of Preventive Medicine* 1999;17(3):176-80. doi: 10.1016/S0749-3797(99)00078-1

13. Sellers TA, Mink PJ, Cerhan JR, et al. The role of hormone replacement therapy in the risk for breast cancer and total mortality in women with a family history of breast cancer. *Annals of internal medicine* 1997;127(11):973-80.

14. White AJ, DeRoo LA, Weinberg CR, et al. Lifetime alcohol intake, binge drinking behaviors, and breast cancer risk. *American Journal of Epidemiology* 2017;186(5):541-49. doi: 10.1093/aje/kwx118

15. Jones ME, Schoemaker MJ, Wright LB, et al. Smoking and risk of breast cancer in the Generations Study cohort. *Breast Cancer Research* 2017;19(1) doi: 10.1186/s13058-017-0908-4

16. Kim HJ, Jung S, Eliassen AH, et al. Alcohol consumption and breast cancer risk in younger women according to family history of breast cancer and folate intake. *American Journal of Epidemiology* 2017;186(5):524-31. doi: 10.1093/aje/kwx137

17. Niehoff NM, Nichols HB, Zhao S, et al. Adult physical activity and breast cancer risk in women with a family history of breast cancer. *Cancer Epidemiology Biomarkers and Prevention* 2019;28(1):51-58. doi: 10.1158/1055-9965.EPI-18-0674

18. Peters TM, Schatzkin A, Gierach GL, et al. Physical activity and postmenopausal breast cancer risk in the NIH-AARP diet and health study. *Cancer Epidemiology Biomarkers and Prevention* 2009;18(1):289-96. doi: 10.1158/1055-9965.EPI-08-0768

19. Sellers TA, Davis J, Cerhan JR, et al. Interaction of waist/hip ratio and family history on the risk of hormone receptor-defined breast cancer in a prospective study of postmenopausal women. *American Journal of Epidemiology* 2002;155(3):225-33. doi: 10.1093/aje/155.3.225

20. Tehard B, Friedenreich CM, Oppert JM, et al. Effect of physical activity on women at increased risk of breast cancer: Results from the E3N cohort study. *Cancer Epidemiology Biomarkers and Prevention* 2006;15(1):57-64. doi: 10.1158/1055-9965.EPI-05-0603

21. Gong Z, Hong CC, Bandera EV, et al. Vigorous physical activity and risk of breast cancer in the African American breast cancer epidemiology and risk consortium. *Breast Cancer Research and Treatment* 2016;159(2):347-56. doi: 10.1007/s10549-016-3936-3

22. Cerhan JR, Grabrick DM, Vierkant RA, et al. Interaction of adolescent anthropometric characteristics and family history on breast cancer risk in a historical cohort study of 426 families (USA). *Cancer Causes and Control* 2004;15(1):1-9. doi: 10.1023/B:CACO.0000016566.30377.4e

23. Couch FJ, Cerhan JR, Vierkant RA, et al. Cigarette smoking increases risk for breast cancer in high-risk breast cancer families. *Cancer Epidemiology Biomarkers and Prevention* 2001;10(4):327-32.

24. Grabrick DM, Hartmann LC, Cerhan JR, et al. Risk of breast cancer with oral contraceptive use in women with a family history of breast cancer. *Journal of the American Medical Association* 2000;284(14):1791-98. doi: 10.1001/jama.284.14.1791

25. Vachon CM, Cerhan JR, Vierkant RA, et al. Investigation of an interaction of alcohol intake and family history on breast cancer risk in the Minnesota breast cancer family study. *Cancer* 2001;92(2):240-48. doi: 10.1002/1097-0142(20010715)92:2<240::AID-CNCR1315>3.0.CO;2-I
