# Supplementary material for: Modifiable risk factors in women at high risk of breast cancer: a systematic review
Source: Breast Cancer Res. 2023 Apr 24;25:45. doi: 10.1186/s13058-023-01636-1 (PMC10123992; doi:10.1186/s13058-023-01636-1)
Supplement: Supplementary file 5 — Additional file 5. Characteristics of included retrospective studies on family history of breast cancer. [file 13058_2023_1636_MOESM5_ESM.docx]

| ADDITIONAL TABLE 2: CHARACTERISTICS OF INCLUDED RETROSPECTIVE STUDIES ON FAMILY HISTORY OF BREAST CANCER | | | | | | | |
| --- | --- | --- | --- | --- | --- | --- | --- |
| Author | Sample Size | Alcohol | Smoking | MHT/hc | Bmi/weight | Physical activity | Notes |
| Bernstein et al. (2005)^1^ | 1225 (775 cases) |  |  |  |  | ✓ |  |
| Grandi et al. (2018)^2^ | 2409 |  |  | ✓ |  |  | 9% of high-risk group and 8% of intermediate-risk group had breast cancer |
| Hirose et al. (2003)^3^ | 973 (157 cases) |  |  |  |  | ✓ |  |
| Marchbanks et al. (2002)^4^ | 1231 (778 cases) |  |  | ✓ |  |  |  |
| Nichols et al. (2007)^5^ | 1446 (427 cases) |  |  | ✓ |  |  |  |
| Patel et al. (2003)^6^ | 171 (110 cases) |  |  |  |  | ✓ |  |
| Sprague et al. (2007)^7^ | 2233 (1238 cases) |  |  |  |  | ✓+  0.91 (0.79-1.05)  (adolescent) |  |
| Murray et al. (1989)^8^ | 834 (554 cases) |  |  | ✓ |  |  |  |
| Newcomb et al. (2002)^9^ | 1968 (1189 cases) |  |  | ✓ |  |  |  |
| Brinton et al. (1986)^10^ | 342 (202 cases) |  | ✓ |  |  |  |  |
| Egan et al. (1998)^11^ | 2292 (1234 cases) | ✓+  2.30, 1.03-5.15  (recent) | ✓ | ✓ | ✓+  1.39 (1.01-1.92)  (gain, POM) | ✓−  0.93 (CI not given)  (strenuous) |  |
| Hirose et al. (2001)^12^ | 739 (109 cases) |  |  |  | ✓+  2.07 (0.6-7.1)  (weight, POM) |  |  |
| La Vecchia et al. (1989) ^13^ | 265 cases | ✓+     (alcohol/day) |  |  |  |  | Number of controls not specified |
| Magnusson et al. (1998)^14^ | 758 (501 cases) |  |  | ✓ | ✓+  2.72 (1.53-4.83)  (BMI, POM) |  |  |
| Peplonska et al. (2008)^15^ | 353 (220 cases) |  |  |  |  | ✓ |  |
| Swerdlow et al. (2002)^16^ | 800 (800 cases) |  |  |  | ✓ | ✓-  0.23 (0.06-0.90) |  |
| Ursin et al. (2002)^17^ | 495 (316 cases) |  |  | ✓ |  |  |  |
| UK National Case-Control Study Group (1990)^18^ | 103 (72 cases) |  |  | ✓ |  |  |  |
| Harris et al. (1990)^19^ | 134 (79 cases) |  |  | ✓ |  |  |  |
| Paul et al. (1990)^20^ | 187 (101 cases) |  |  | ✓ |  |  |  |
| Tavani et al. (1993)^21^ | 320 (245 cases) |  |  | ✓ |  |  |  |
| No author listed (Division of Reproductive Health, Centers for Disease Control) (1983)^22^ | 152 (87 cases) |  |  | ✓ |  |  |  |
| Claus et al. (2003)^23^ | 148 (83 cases) |  |  | ✓ |  |  |  |
| Rohan et al. (1988)^24^ | 61 (37 cases) |  |  | ✓ |  |  |  |
| Nomura et al. (1986)^25^ | 57 (38 cases) |  |  | ✓ |  |  |  |
| Brinton et al. (1979)^26^ | 170 (78 cases) |  |  | ✓ |  |  |  |
| Carpenter et al. (2003)^27^ | 495 (316 cases) |  |  |  | ✓+  2.90 (1.86-4.54)  (BMI, POM)  3.03 (1.95-4.71)  (gain, POM) | ✓ |  |
| Dinger et al. (2006)^28^ | 1199 (550 cases) |  |  | ✓+  6.4 (1.7–24.5)  (MHT, ever) |  |  |  |
| Katsouyanni et al. (1997)^29^ | 112 (43 cases) | ✓ |  | ✓ | ✓ |  |  |
| Suzuki et al. (2007)^30^ | 1680 |  | ✓+  4.33 (1.65-11.40)   |  |  |  | Number of cases and controls with FH not specified |
| Verloop et al. (2000)^31^ | 176 (119 cases) |  |  |  |  | ✓-  0.28 (0.14-0.58) |  |
| Brinton et al. (1982)^32^ | 69 (48 cases) |  |  | ✓ |  |  |  |
| White et al. (1994)^33^ | 168 (119 cases) |  |  | ✓ |  |  |  |
| Ravnihar et al. (1988)^34^ | 24 (14 cases) |  |  | ✓+  7.36 (3.09-17.52)  (HC, ever) |  |  |  |
| Pesch et al. (2005)^35^ | 30 (15 cases) |  |  | ✓+  1.05 (1.02-1.47)  (MHT, in menopause) |  |  |  |
| Huang et al. (2004)^36^ | 1487 (146 cases) | ✓ | ✓ |  |  | ✓-  0.61 (0.39-0.96)  (current |  |
| Toss et al. (2017)^37^ | 2409 (210 cases) |  |  | ✓ |  |  |  |
| Park et al. (2017)^38^ | 1038 (882 cases) |  |  | ✓ |  |  | Study reports 1083 but numbers add up to 1038 |
| ✓Study presented data on the association of the modifiable risk factor with breast cancer. Association presented was positive (+), negative (-), or not significant (no +/- listed). Associations could be both (+) and (–) if multiple associations were presented.  Significant risk estimates (RR/OR/HR (95% CI)) from studies are listed. Results from studies reporting only p-values or other measures that did not indicate magnitude of effect are not included in this table. | | | | | | | |

**References**

1. Bernstein L, Patel AV, Ursin G, et al. Lifetime recreational exercise activity and breast cancer risk among black women and white women. *Journal of the National Cancer Institute* 2005;97(22):1671-79. doi: 10.1093/jnci/dji374

2. Grandi G, Toss A, Cagnacci A, et al. Combined Hormonal Contraceptive Use and Risk of Breast Cancer in a Population of Women With a Family History. *Clinical Breast Cancer* 2018;18(1):e15-e24. doi: 10.1016/j.clbc.2017.10.016

3. Hirose K, Hamajima N, Takezaki T, et al. Physical exercise reduces risk of breast cancer in Japanese women. *Cancer Science* 2003;94(2):193-99. doi: 10.1111/j.1349-7006.2003.tb01418.x

4. Marchbanks PA, McDonald JA, Wilson HG, et al. Oral contraceptives and the risk of breast cancer. *The New England journal of medicine* 2002;346(26):2025-32.

5. Nichols HB, Trentham-Dietz A, Egan KM, et al. Oral contraceptive use and risk of breast carcinoma in situ. *Cancer epidemiology, biomarkers & prevention : a publication of the American Association for Cancer Research, cosponsored by the American Society of Preventive Oncology* 2007;16(11):2262-8.

6. Patel AV, Press MF, Meeske K, et al. Lifetime Recreational Exercise Activity and Risk of Breast Carcinoma In Situ. *Cancer* 2003;98(10):2161-69. doi: 10.1002/cncr.11768

7. Sprague BL, Trentham-Dietz A, Newcomb PA, et al. Lifetime recreational and occupational physical activity and risk of in situ and invasive breast cancer. *Cancer Epidemiology Biomarkers and Prevention* 2007;16(2):236-43. doi: 10.1158/1055-9965.EPI-06-0713

8. Murray PP, Stadel BV, Schlesselman JJ. Oral contraceptive use in women with a family history of breast cancer. *Obstetrics and gynecology* 1989;73(6):977-83.

9. Newcomb PA, Titus-Ernstoff L, Egan KM, et al. Postmenopausal estrogen and progestin use in relation to breast cancer risk. *Cancer Epidemiology Biomarkers and Prevention* 2002;11(7):593-600.

10. Brinton LA, Schairer C, Stanford JL, et al. Cigarette smoking and breast cancer. *American journal of epidemiology* 1986;123(4):614-22.

11. Egan KM, Stampfer MJ, Rosner BA, et al. Risk factors for breast cancer in women with a breast cancer family history. *Cancer Epidemiology Biomarkers and Prevention* 1998;7(5):359-64.

12. Hirose K, Tajima K, Hamajima N, et al. Association of family history and other risk factors with breast cancer risk among Japanese premenopausal and postmenopausal women. *Cancer Causes and Control* 2001;12(4):349-58. doi: 10.1023/A:1011232602348

13. La Vecchia A, Negri E, Parazzini F, et al. Alcohol and breast cancer: Update from an Italian case-control study. *European Journal of Cancer and Clinical Oncology* 1989;25(12):1711-17. doi: 10.1016/0277-5379(89)90339-8

14. Magnusson C, Colditz G, Rosner B, et al. Association of family history and other risk factors with breast cancer risk (Sweden). *Cancer Causes and Control* 1998;9(3):259-67. doi: 10.1023/A:1008817018942

15. Peplonska B, Lissowska J, Hartman TJ, et al. Adulthood lifetime physical activity and breast cancer. *Epidemiology* 2008;19(2):226-36. doi: 10.1097/EDE.0b013e3181633bfb

16. Swerdlow AJ, De Stavola BL, Floderus B, et al. Risk factors for breast cancer at young ages in twins: An international population-based study. *Journal of the National Cancer Institute* 2002;94(16):1238-46.

17. Ursin G, Tseng CC, Paganini-Hill A, et al. Does menopausal hormone replacement therapy interact with known factors to increase risk of breast cancer? *Journal of Clinical Oncology* 2002;20(3):699-706. doi: 10.1200/JCO.20.3.699

18. Oral contraceptive use and breast cancer risk in young women: Subgroup analyses. *Lancet* 1990;335(8704):1507-09. doi: 10.1016/0140-6736(90)93038-Q

19. Harris RE, Zang EA, Wynder EL. Oral contraceptives and breast cancer risk: a case-control study. *International journal of epidemiology* 1990;19(2):240-6.

20. Paul C, Skegg DC, Spears GF. Oral contraceptives and risk of breast cancer. *International journal of cancer* 1990;46(3):366-73.

21. Tavani A, Negri E, Franceschi S, et al. Oral contraceptives and breast cancer in northern Italy. Final report from a case-control study. *British journal of cancer* 1993;68(3):568-71.

22. Long-term oral contraceptive use and the risk of breast cancer. The centers for disease control cancer and steroid hormone study. *Journal of the American Medical Association* 1983;249(12):1591-95. doi: 10.1001/jama.249.12.1591

23. Claus EB, Stowe M, Carter D. Oral contraceptives and the risk of ductal breast carcinoma in situ. *Breast cancer research and treatment* 2003;81(2):129-36.

24. Rohan TE, McMichael AJ. Oral contraceptive agents and breast cancer: a population-based case-control study. *The Medical journal of Australia* 1988;149(10):520-6.

25. Nomura AM, Kolonel LN, Hirohata T, et al. The association of replacement estrogens with breast cancer. *International journal of cancer* 1986;37(1):49-53.

26. Brinton LA, Williams RR, Hoover RN. Breast cancer risk factors among screening program participants. *Journal of the National Cancer Institute* 1979;62(1):37-44.

27. Carpenter CL, Ross RK, Paganini-Hill A, et al. Effect of family history, obesity and exercise on breast cancer risk among postmenopausal women. *International Journal of Cancer* 2003;106(1):96-102. doi: 10.1002/ijc.11186

28. Dinger JC, Heinemann LAJ, Möhner S, et al. Breast cancer risk associated with different MHT formulations: A register-based case-control study. *BMC Women's Health* 2006;6 doi: 10.1186/1472-6874-6-13

29. Katsouyanni K, Signorello LB, Lagiou P, et al. Evidence that adult life risk factors influence the expression of familial propensity to breast cancer. *Epidemiology (Cambridge, Mass)* 1997;8(5):592-5.

30. Suzuki T, Matsuo K, Wakai K, et al. Effect of familial history and smoking on common cancer risks in Japan. *Cancer* 2007;109(10):2116-23. doi: 10.1002/cncr.22685

31. Verloop J, Rookus MA, van der Kooy K, et al. Physical activity and breast cancer risk in women aged 20-54 years. *J Natl Cancer Inst* 2000;92(2):128-35. doi: 10.1093/jnci/92.2.128 [published Online First: 2000/01/20]

32. Brinton LA, Hoover R, Szklo M, et al. Oral contraceptives and breast cancer. *International journal of epidemiology* 1982;11(4):316-22.

33. White E, Malone KE, Weiss NS, et al. Breast cancer among young U.S. women in relation to oral contraceptive use. *Journal of the National Cancer Institute* 1994;86(7):505-14.

34. Ravnihar B, Primic Zakelj M, Kosmelj K, et al. A case-control study of breast cancer in relation to oral contraceptive use in Slovenia. *Neoplasma* 1988;35(1):109-21.

35. Pesch B, Ko Y, Brauch H, et al. Factors modifying the association between hormone-replacement therapy and breast cancer risk. *European journal of epidemiology* 2005;20(8):699-711.

36. Huang XE, Hirose K, Wakai K, et al. Comparison of lifestyle risk factors by family history for gastric, breast, lung and colorectal cancer. *Asian Pacific journal of cancer prevention : APJCP* 2004;5(4):419-27.

37. Toss A, Grandi G, Cagnacci A, et al. The impact of reproductive life on breast cancer risk in women with family history or BRCA mutation. *Oncotarget* 2017;8(6):9144-54. doi: <https://dx.doi.org/10.18632/oncotarget.13423>

38. Park B, Hopper JL, Win AK, et al. Reproductive factors as risk modifiers of breast cancer in BRCA mutation carriers and high-risk non-carriers. *Oncotarget* 2017;8(60):102110-18. doi: <https://dx.doi.org/10.18632/oncotarget.22193>
