# Supplementary material for: Modifiable risk factors in women at high risk of breast cancer: a systematic review
Source: Breast Cancer Res. 2023 Apr 24;25:45. doi: 10.1186/s13058-023-01636-1 (PMC10123992; doi:10.1186/s13058-023-01636-1)
Supplement: Supplementary file 6 — Additional file 6. Full search strategy. [file 13058_2023_1636_MOESM6_ESM.docx]

Nov 13, 2019 with addition of italicized
Total: 1,859
Duplicates: 766
Unique: 1,093

July 2019
Total: 11,710
Duplicates: 5,622
Unique: 6,792

**Ovid Medline**

07/29/19
2706 results

11/12/19
734 results with addition of italicized

**Breast Cancer**

Exp breast neoplasms/ OR ((breast OR mamma OR mammary OR lobular) adj2 (Cancer* or carcinoma* or neoplas* or tumor* or tumour* or malignan*)).mp.

**High Risk for Breast Cancer**

Exp genetic predisposition to disease/ OR Exp Neoplasm Genes/ OR Exp Li-Fraumeni Syndrome/ OR Exp PTEN Phosphohydrolase/ OR Exp multiple Hamartoma Syndrome/ OR Exp Peutz-Jeghers Syndrome/ OR Exp Ataxia Telangiectasia/ OR familial.mp. OR (genetic adj1 (predisposition OR susceptibility)).mp. OR Family history.mp. OR hereditary.mp. OR genetic predisposition.mp. OR BRCA*.mp. OR Li Fraumeni.mp. OR cowden*.mp. OR multiple hamartoma syndrome.mp. OR PTEN Protein.mp. OR Lhermitte Duclos.mp. OR Cerebellum Dysplastic Gangliocytoma.mp. OR PTEN hamartoma.mp. OR Bannayan Riley Ruvalcaba.mp. OR Ruvalcaba Myhre.mp. OR myhre riley smith.mp. OR ruvalcaba myhre smith.mp. OR bannayan ruvalcaba riley.mp. OR bannayan zonana.mp. OR multiple hemangiomata.mp. OR (PTEN adj3 hamartoma).mp. OR Peutz jehgers.mp. OR (Hamartomatous adj2 Polyp*).mp. OR (Perior* adj1 Lentiginos*).mp. OR "polyps and spots syndrome".mp. OR Ataxia Telangiectasia.mp. OR louis bar syndrome.mp. OR louis bar disease.mp. OR telangiectasia cerebellooculocutanea.mp. OR Breast Cancer Type 1 Susceptibility Protein.mp. OR breast cancer 1 protein.mp. OR ring finger protein 53.mp. OR breast cancer 1 gene product.mp. OR inherited cancer syndrome cell line.mp.

**Modifiable Risk Factors**

Exp Exercise/ OR Exp diet/ OR Exp smoking/ OR Exp smoking cessation/ OR Exp alcohol drinking/ OR Exp alcoholic beverages/ OR Exp body weight/ OR Exp body mass index/ OR Exp waist-hip ratio/ OR Exp body fat distribution/ OR Exp sedentary behavior/ OR Exp life style/ OR modifiable.mp. OR body fat distribution.mp. OR adiposity.mp. OR bmi.mp. OR body mass index.mp. OR weight.mp. OR overweight.mp. OR obesity.mp. OR obese.mp. OR alcohol.mp. OR smoking.mp. OR nicotine.mp. OR diet.mp. OR exercise.mp. OR Physical activity.mp. OR physical inactivity.mp. OR sedentary.mp. OR life style.mp. OR lifestyle*.mp. OR vaping.mp.

OR

Exp oral contraceptives/ OR Exp postcoital contraceptives/ OR Exp Intrauterine devices/ OR (intrauterine adj2 (device* OR coil)).mp. OR IUD.mp. OR ((hormonal OR oral OR implant* OR agent* OR injectable OR injection* OR device* OR coil) adj2 (contraception OR contraceptive*)).mp. OR (vaginal adj1 ring*).mp. OR (birth adj1 control).mp. OR norplant.mp. OR implanon.mp. OR nexplanon.mp. OR depoprovera.mp. OR (depo adj2 provera).mp.

OR
*Exp hormone replacement therapy/ OR hormone replacement therapy.mp. OR hormone replacement therapies.mp. OR estrogen replacement therapy.mp. OR ((oestrogen OR estrogen) adj2 (replacement OR therap* OR substitution)).mp. OR hormone substitution.mp. OR Estrogen Progestin Combination Therapy.mp.*

**Embase**

07/29/19
5,579

11/13/19
427

**Breast Cancer**

‘breast cancer’/exp OR ((breast OR mamma OR mammary OR lobular) near/2 (Cancer* or carcinoma* or neoplas* or tumor* or tumour* or malignan*)):ti,ab,kw,de

**High Risk for Breast Cancer**

'genetic predisposition'/exp OR 'BRCA1 protein'/exp OR 'BRCA2 protein'/exp OR 'hereditary tumor syndrome cell line'/exp OR 'Li-Fraumeni syndrome'/exp OR 'phosphatidylinositol 3,4,5 trisphosphate 3 phosphatase'/exp OR 'Cowden syndrome'/exp OR ‘peutz jeghers syndrome’/exp OR 'ataxia telangiectasia'/exp OR familial:ti,ab,kw OR (genetic near/1 (predisposition OR susceptibility)):ti,ab,kw,de OR ‘Family history’:ti,ab,kw,de OR hereditary:ti,ab,kw OR ‘genetic predisposition’:ti,ab,kw,de OR BRCA*:ti,ab,kw,de OR ‘Li Fraumeni’:ti,ab,kw,de OR cowden*:ti,ab,kw,de OR ‘multiple hamartoma syndrome’:ti,ab,kw,de OR ‘PTEN Protein’:ti,ab,kw,de OR ‘Lhermitte Duclos’:ti,ab,kw,de OR ‘Cerebellum Dysplastic Gangliocytoma’:ti,ab,kw,de OR ‘PTEN hamartoma’:ti,ab,kw,de OR ‘Bannayan Riley Ruvalcaba’:ti,ab,kw,de OR ‘Ruvalcaba Myhre’:ti,ab,kw,de OR ‘myhre riley smith’:ti,ab,kw,de OR ‘ruvalcaba myhre smith’:ti,ab,kw,de OR ‘bannayan ruvalcaba riley’:ti,ab,kw,de OR ‘bannayan zonana’:ti,ab,kw,de OR ‘multiple hemangiomata’:ti,ab,kw,de OR (PTEN near/3 hamartoma):ti,ab,kw,de OR ‘Peutz jehgers’:ti,ab,kw,de OR (Hamartomatous near/2 Polyp*):ti,ab,kw,de OR (Perior* near/1 Lentiginos*):ti,ab,kw,de OR ‘polyps and spots syndrome’:ti,ab,kw,de OR ‘Ataxia Telangiectasia’:ti,ab,kw,de OR ‘louis bar syndrome’:ti,ab,kw,de OR ‘louis bar disease’:ti,ab,kw,de OR ‘telangiectasia cerebellooculocutanea’:ti,ab,kw,de OR ‘Breast Cancer Type 1 Susceptibility Protein’:ti,ab,kw,de OR ‘breast cancer 1 protein’:ti,ab,kw,de OR ‘ring finger protein 53’:ti,ab,kw,de OR ‘breast cancer 1 gene product’:ti,ab,kw,de OR ‘inherited cancer syndrome cell line’:ti,ab,kw,de

**Modifiable Risk Factors**

‘Exercise’/exp OR ‘physical activity’/exp OR ‘diet’/exp OR ‘smoking’/exp OR ‘smoking cessation’/exp OR ‘drinking behavior’/exp OR ‘alcoholic beverage’/exp OR ‘body weight’/exp OR ‘body mass’/exp OR ‘waist hip ratio’/exp OR ‘waist to height ratio’/exp OR ‘weight height ratio’/exp OR ‘fat mass’/exp OR ‘body fat distribution’/exp OR ‘sedentary lifestyle’/exp OR ‘lifestyle’/exp OR modifiable:ti,ab,kw OR ‘body fat distribution’:ti,ab,kw,de OR adiposity:ti,ab,kw,de OR bmi:ti,ab,kw,de OR ‘body mass index’:ti,ab,kw,de OR weight:ti,ab,kw,de OR overweight:ti,ab,kw,de OR obesity:ti,ab,kw,de OR obese:ti,ab,kw,de OR alcohol:ti,ab,kw OR smoking:ti,ab,kw,de OR nicotine:ti,ab,kw,de OR diet:ti,ab,kw,de OR exercise:ti,ab,kw,de OR ‘Physical activity’:ti,ab,kw,de OR ‘physical inactivity’:ti,ab,kw,de OR sedentary:ti,ab,kw,de OR ‘life style’:ti,ab,kw,de OR lifestyle*:ti,ab,kw,de OR vaping:ti,ab,kw,de

OR

‘contraceptive agent’/exp OR 'intrauterine contraceptive device'/exp OR (intrauterine near/2 (device* OR coil)):ti,ab,kw,de OR IUD:ti,ab,kw,de OR ((hormonal OR oral OR implant* OR agent* OR injectable OR injection* OR device* OR coil) near/2 (contraception OR contraceptive*)):ti,ab,kw,de OR (vaginal near/1 ring*):ti,ab,kw,de OR (birth near/1 control):ti,ab,kw,de OR norplant:ti,ab,kw,de OR implanon:ti,ab,kw,de OR nexplanon:ti,ab,kw,de OR depoprovera:ti,ab,kw,de OR (depo near/2 provera):ti,ab,kw,de

OR
*‘hormone substitution’/exp OR ‘hormone replacement therapy’:ti,ab,kw,de OR ‘hormone replacement therapies’:ti,ab,kw,de OR ‘estrogen replacement therapy’:ti,ab,kw,de OR ((oestrogen OR estrogen) near/2 (replacement OR therap* OR substitution)):ti,ab,kw,de OR ‘hormone substitution’:ti,ab,kw,de OR ‘Estrogen Progestin Combination Therapy’:ti,ab,kw,de*

**Cochrane Central**

06/27/19
161 results

07/29/19
171 results

**Breast Cancer**

[mh “breast neoplasms”] OR ((breast OR mamma OR mammary OR lobular) near/2 (Cancer* or carcinoma* or neoplas* or tumor* or tumour* or malignan*)):ti,ab,kw

**High Risk for Breast Cancer**

[mh "genetic predisposition to disease"] OR [mh "Neoplasm Genes"] OR [mh "Li-Fraumeni Syndrome"] OR [mh "PTEN Phosphohydrolase"] OR [mh "multiple Hamartoma Syndrome"] OR [mh "Peutz-Jeghers Syndrome"] OR [mh "Ataxia Telangiectasia"] OR familial:ti,ab,kw OR (genetic near/1 (predisposition OR susceptibility)):ti,ab,kw OR “Family history”:ti,ab,kw OR hereditary:ti,ab,kw OR “genetic predisposition”:ti,ab,kw OR BRCA*:ti,ab,kw OR “Li Fraumeni”:ti,ab,kw OR cowden*:ti,ab,kw OR “multiple hamartoma syndrome”:ti,ab,kw OR “PTEN Protein”:ti,ab,kw OR “Lhermitte Duclos”:ti,ab,kw OR “Cerebellum Dysplastic Gangliocytoma”:ti,ab,kw OR “PTEN hamartoma”:ti,ab,kw OR “Bannayan Riley Ruvalcaba”:ti,ab,kw OR “Ruvalcaba Myhre”:ti,ab,kw OR “myhre riley smith”:ti,ab,kw OR “ruvalcaba myhre smith”:ti,ab,kw OR “bannayan ruvalcaba riley”:ti,ab,kw OR “bannayan zonana”:ti,ab,kw OR “multiple hemangiomata”:ti,ab,kw OR (PTEN near/3 hamartoma):ti,ab,kw OR “Peutz jehgers”:ti,ab,kw OR (Hamartomatous near/2 Polyp*):ti,ab,kw OR (Perior* near/1 Lentiginos*):ti,ab,kw OR “polyps and spots syndrome”:ti,ab,kw OR “Ataxia Telangiectasia”:ti,ab,kw OR “louis bar syndrome”:ti,ab,kw OR “louis bar disease”:ti,ab,kw OR “telangiectasia cerebellooculocutanea”:ti,ab,kw OR “Breast Cancer Type 1 Susceptibility Protein”:ti,ab,kw OR “breast cancer 1 protein”:ti,ab,kw OR “ring finger protein 53”:ti,ab,kw OR “breast cancer 1 gene product”:ti,ab,kw OR “inherited cancer syndrome cell line”:ti,ab,kw

**Modifiable Risk Factors**

[mh exercise] OR [mh diet] or [mh smoking] OR [mh "smoking cessation"] OR [mh "alcohol drinking"] OR [mh "alcoholic beverages"] OR [mh "body weight"] OR [mh "body mass index"] OR [mh "waist-hip ratio"] OR [mh "body fat distribution"] OR [mh "sedentary behavior"] OR [mh "life style"] OR modifiable:ti,ab,kw OR “body fat distribution”:ti,ab,kw OR adiposity:ti,ab,kw OR bmi:ti,ab,kw OR “body mass index”:ti,ab,kw OR weight:ti,ab,kw OR overweight:ti,ab,kw OR obesity:ti,ab,kw OR obese:ti,ab,kw OR alcohol:ti,ab,kw OR smoking:ti,ab,kw OR nicotine:ti,ab,kw OR diet:ti,ab,kw OR exercise:ti,ab,kw OR “Physical activity”:ti,ab,kw OR “physical inactivity”:ti,ab,kw OR sedentary:ti,ab,kw OR “life style”:ti,ab,kw OR lifestyle*:ti,ab,kw OR vaping:ti,ab,kw OR [mh "oral contraceptives"] OR [mh "postcoital contraceptives"] OR [mh "Intrauterine devices"]
OR

*[mh “hormone replacement therapy”] OR “hormone replacement therapy”:ti,ab,kw OR “hormone replacement therapies”:ti,ab,kw OR “estrogen replacement therapy”:ti,ab,kw OR ((oestrogen OR estrogen) near/2 (replacement OR therap* OR substitution)):ti,ab,kw OR “hormone substitution”:ti,ab,kw OR “Estrogen Progestin Combination Therapy”:ti,ab,kw*

**SCOPUS**

07/29/19
3252 results

11/13/19
550 results + 148 results

( ( TITLE-ABS-KEY ( genetic W/1 ( predisposition OR susceptibility ) ) ) OR ( TITLE-ABS-KEY ( "Family history" ) ) OR ( TITLE ( familial ) ) OR ( TITLE ( hereditary ) ) OR ( TITLE-ABS-KEY ( "genetic predisposition" ) ) OR ( TITLE-ABS-KEY ( brca* ) ) OR ( TITLE-ABS-KEY ( "Li Fraumeni" ) ) OR ( TITLE-ABS-KEY ( cowden* ) ) OR ( TITLE-ABS-KEY ( "multiple hamartoma syndrome" ) ) OR ( TITLE-ABS-KEY ( "PTEN Protein" ) ) OR ( TITLE-ABS-KEY ( "Lhermitte Duclos" ) ) OR ( TITLE-ABS-KEY ( "Cerebellum Dysplastic Gangliocytoma" ) ) OR ( TITLE-ABS-KEY ( "PTEN hamartoma" ) ) OR ( TITLE-ABS-KEY ( "Bannayan Riley Ruvalcaba" ) ) OR ( TITLE-ABS-KEY ( "Ruvalcaba Myhre" ) ) OR ( TITLE-ABS-KEY ( "myhre riley smith" ) ) OR ( TITLE-ABS-KEY ( "ruvalcaba myhre smith" ) ) OR ( TITLE-ABS-KEY ( "bannayan ruvalcaba riley" ) ) OR ( TITLE-ABS-KEY ( "bannayan zonana" ) ) OR ( TITLE-ABS-KEY ( "multiple hemangiomata" ) ) OR ( TITLE-ABS-KEY ( pten W/3 hamartoma ) ) OR ( TITLE-ABS-KEY ( "Peutz jehgers" ) ) OR ( TITLE-ABS-KEY ( hamartomatous W/2 polyp* ) ) OR ( TITLE-ABS-KEY ( perior* W/1 lentiginos* ) ) OR ( TITLE-ABS-KEY ( "polyps and spots syndrome" ) ) OR ( TITLE-ABS-KEY ( "Ataxia Telangiectasia" ) ) OR ( TITLE-ABS-KEY ( "louis bar syndrome" ) ) OR ( TITLE-ABS-KEY ( "louis bar disease" ) ) OR ( TITLE-ABS-KEY ( "telangiectasia cerebellooculocutanea" ) ) OR ( TITLE-ABS-KEY ( "Breast Cancer Type 1 Susceptibility Protein" ) ) OR ( TITLE-ABS-KEY ( "breast cancer 1 protein" ) ) OR ( TITLE-ABS-KEY ( "ring finger protein 53" ) ) OR ( TITLE-ABS-KEY ( "breast cancer 1 gene product" ) ) OR ( TITLE-ABS-KEY ( "inherited cancer syndrome cell line" ) ) ) AND ( ( TITLE ( modifiable ) ) OR ( TITLE-ABS-KEY ( "body fat distribution" ) ) OR ( TITLE-ABS-KEY ( adiposity ) ) OR ( TITLE ( bmi ) ) OR ( TITLE-ABS-KEY ( "body mass index" ) ) OR ( TITLE ( weight ) ) OR ( TITLE-ABS-KEY ( overweight ) ) OR ( TITLE-ABS-KEY ( obesity ) ) OR ( TITLE-ABS-KEY ( obese ) ) OR ( TITLE ( alcohol ) ) OR ( TITLE-ABS-KEY ( smoking ) ) OR ( TITLE-ABS-KEY ( nicotine ) ) OR ( TITLE ( diet ) ) OR ( TITLE ( dieting ) ) OR ( TITLE ( diets ) ) OR ( TITLE ( exercis* ) ) OR ( TITLE-ABS-KEY ( "Physical activity" ) ) OR ( TITLE-ABS-KEY ( "physical inactivity" ) ) OR ( TITLE-ABS-KEY ( sedentary ) ) OR ( TITLE ( "life style" ) ) OR ( TITLE ( lifestyle* ) ) OR ( TITLE-ABS-KEY ( vaping ) ) OR ( TITLE-ABS-KEY ( intrauterine W/2 ( device* OR coil ) ) ) OR ( TITLE-ABS-KEY ( iud ) ) OR ( TITLE-ABS-KEY ( ( hormonal OR oral OR implant* OR agent* OR injectable OR injection* OR device* OR coil ) W/2 ( contraception OR contraceptive* ) ) ) OR ( TITLE-ABS-KEY ( vaginal W/1 ring* ) ) OR ( TITLE-ABS-KEY ( birth W/1 control ) ) OR ( TITLE-ABS-KEY ( norplant ) ) OR ( TITLE-ABS-KEY ( implanon ) ) OR ( TITLE-ABS-KEY ( nexplanon ) ) OR ( TITLE-ABS-KEY ( depoprovera ) ) OR ( TITLE-ABS-KEY ( depo W/2 provera ) ) ) AND ( ( TITLE-ABS-KEY ( breast W/2 ( cancer* OR carcinoma* OR neoplas* OR tumor* OR tumour* OR malignan* ) ) ) )

*(TITLE-ABS-KEY (“hormone replacement therapy”)) OR (TITLE-ABS-KEY ( “hormone replacement therapies”)) OR (TITLE-ABS-KEY ( “estrogen replacement therapy”)) OR (TITLE-ABS-KEY ( oestrogen OR estrogen) W/2 (replacement OR therap* OR substitution))) OR (TITLE-ABS-KEY ( “hormone substitution”)) OR (TITLE-ABS-KEY ( “Estrogen Progestin Combination Therapy”))*

**Clinicaltrials.gov**

2 results

( BRCA1 OR BRCA2 OR "family history" OR "genetic predispotition" ) AND INFLECT EXACT "Completed" [OVERALL-STATUS] AND Breast Cancer [DISEASE] AND ( smoking cessation OR diet OR physical activity OR body mass index OR exercise OR oral contraceptives *OR hormone replacement therapy* ) [TREATMENT]
